# The Production of Curli Amyloid Fibers Is Deeply Integrated into the Biology of *Escherichia coli*

**DOI:** 10.3390/biom7040075

**Published:** 2017-10-31

**Authors:** Daniel R. Smith, Janet E. Price, Peter E. Burby, Luz P. Blanco, Justin Chamberlain, Matthew R. Chapman

**Affiliations:** Department of Molecular, Cellular, and Developmental Biology, University of Michigan, Ann Arbor, MI 48109, USA; dnlrsmith@gmail (D.R.S.); janetep@umich.edu (J.E.P.); pburby@umich.edu (P.E.B.); luz.blanco@gmail.com (L.P.B.); justcham@umich.edu (J.C.)

**Keywords:** amyloid, curli, Keio collection, csgA, csgD, LPS, RpoS, RpoE, *Escherichia coli*, nhaA, rafF

## Abstract

Curli amyloid fibers are the major protein component of the extracellular matrix produced by Enterobacteriaceae during biofilm formation. Curli are required for proper biofilm development and environmental persistence by *Escherichia coli*. Here, we present a complete and vetted genetic analysis of functional amyloid fiber biogenesis. The Keio collection of single gene deletions was screened on Congo red indicator plates to identify *E. coli* mutants that had defective amyloid production. We discovered that more than three hundred gene products modulated curli production. These genes were involved in fundamental cellular processes such as regulation, environmental sensing, respiration, metabolism, cell envelope biogenesis, transport, and protein turnover. The alternative sigma factors, σ^S^ and σ^E^, had opposing roles in curli production. Mutations that induced the σ^E^ or Cpx stress response systems had reduced curli production, while mutant strains with increased σ^S^ levels had increased curli production. Mutations in metabolic pathways, including gluconeogenesis and the biosynthesis of lipopolysaccharide (LPS), produced less curli. Regulation of the master biofilm regulator, CsgD, was diverse, and the screen revealed several proteins and small RNAs (sRNA) that regulate *csgD* messenger RNA (mRNA) levels. Using previously published studies, we found minimal overlap between the genes affecting curli biogenesis and genes known to impact swimming or swarming motility, underlying the distinction between motile and sessile lifestyles. Collectively, the diversity and number of elements required suggest curli production is part of a highly regulated and complex developmental pathway in *E. coli*.

## 1. Introduction

Curli are thin aggregative fimbriae produced by many Enterobacteriaceae including *Escherichia coli* and *Salmonella typhimurium* [1,2]. As the major protein component of the extracellular matrix, curli are associated with biofilm development [3]. Furthermore, curliated bacteria adhere to and colonize a variety of surfaces [4,5,6,7,8] and are resistant to damage from predation, desiccation, oxidative stress, and other antimicrobial agents [4,5,9,10,11]. Curli fibers have also been implicated in pathogenesis and aid in host cell adhesion, invasion, and immune system activation [12,13,14].

Curli fibers are the product of a dedicated and highly regulated assembly system encoded by curli specific genes (*csg*), which are arranged on two divergent operons *csgDEFG* and *csgBAC* [15,16,17]. Found primarily in the genomes of Enterobacteriaceae, *csg* genes are also present in other Gammaproteobacteria including members of the Pseudomonadaceae, Shewanellaceae, and Vibrionaceae families, as well as the more distantly related Bradyrhizobiaceae, Burkholderiaceae, and Flavobacteriaceae families [18]. CsgD is the master biofilm regulator, and is required for transcription of the *csgBAC* operon [15,19]. At the cell surface, the major curli fiber subunit CsgA is nucleated into an extracellular amyloid fiber by the minor fiber subunit CsgB [1,20,21,22]. Secretion of both curli fiber subunits requires the lipoprotein CsgG, which forms a pore within the outer membrane [1,23,24,25,26]. CsgE acts as the cap for CsgG, blocking the unfolded CsgA from returning into the periplasm [27]. CsgF has been shown to interact with CsgG and may aid in its function [25,26,28], as well as keeping CsgB associated with the bacterial outer membrane [22,29]. CsgC is dispensable for curli formation; however, it may influence curli fiber morphology, and it potently discourages CsgA amyloid formation within the cell [15,30,31,32]. As the first bacterial protein identified as an amyloid [1], curli are part of a growing class of functional amyloid proteins that have evolved to fulfill physiological roles [33]. 

Regulation of *csgDEFG* operon is complex, and involves several proteins [16]. Transcription of the *csgDEFG* operon is under the control of RpoS (σ^S^), Crl, MlrA, H-NS, IHF, and a number of bacterial two component systems [16,34] (see Table S4). The intervening region between the curli operons is the fifth largest divergent operon in *E. coli*, yet previous screens to find additional accessory factors and regulators have identified only a handful of genes including *nagA*, *ompR*, *dksA*, and *rssB* [15,35,36]. The current cohort of regulators does not sufficiently account for the diverse conditions that induce *E. coli* expression of curli and other biofilm components [37,38,39]. Therefore, we performed a comprehensive screen for genes involved in curli production using the Keio collection [40]. The Keio collection is a near complete set of single gene knockouts in K-12 *E. coli*. By growing the collection on plates containing the amyloid specific dye Congo red (CR), we identified more than 300 strains that had changes in curli production. These strains lack genes involved in a variety of cellular processes including regulation, environmental sensing, respiration, metabolism, cell envelope biogenesis, transport, and protein turnover. Many of these genes are known to affect RpoS (σ^S^) levels or induce the Cpx or RpoE (σ^E^) stress responses. Induction of either the Cpx or σ^E^ stress response system resulted in less curli production, while stimulation of σ^S^ levels increased curli expression. Few of the genes identified here are involved in motility, underlying the fact that motility and biofilm formation are distinct microbial behaviors. We propose that curli fiber formation is part of a unique lifestyle in *E. coli* that is tightly linked to key metabolic pathways, including nucleotide synthesis, cell envelope maintenance, and the citric acid cycle.

## 2. Results and Discussion

### 2.1. Forward Genetic Screen to Identify Genes Involved in Curli Production

**Figure 1:**
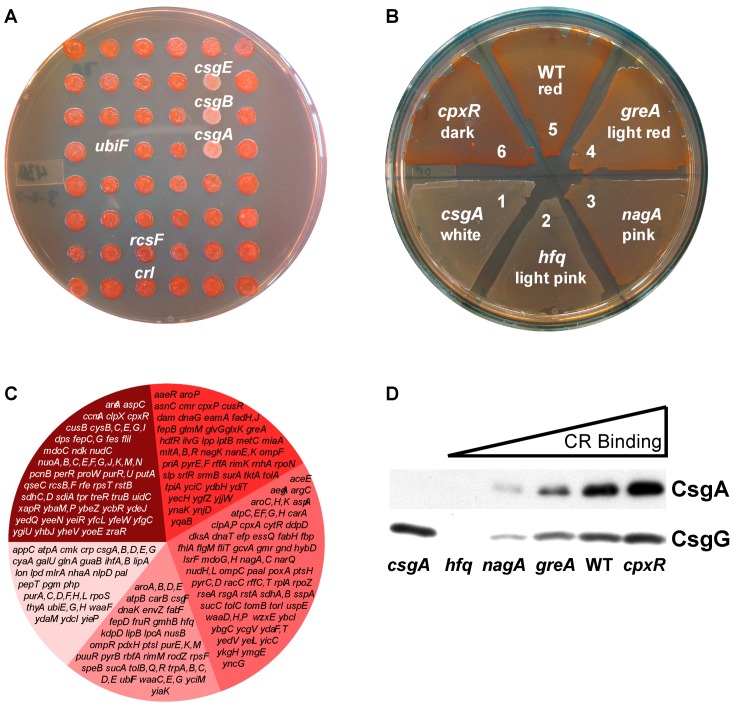
Overview of screen for mutants affecting curli production. (**A**) The Keio collection was screened on CR indicator plates after inoculation with a bolt replicator and incubation for two days at 26 °C. The collection was screened three times. (**B**) Associated phenotype scoring: 1 or white = *csgA*; 2 or light pink = *hfq*; 3 or pink = *nagA*; 4 or light red = *greA*; 5 or wild type (WT) = BW25113; and 6 or dark red = *cpxR*. (**C**) Distribution of Congo red (CR) phenotypes based on scoring from white to dark red. (**D**) Whole cell Western blots of strains probed with anti-CsgG and anti-CsgA antibodies.

To better understand curli production and regulation, we screened the Keio collection for mutants with altered curli production using Congo red (CR) indicator plates (Figure 1A,B). Curliated colonies turn red when grown on CR plates, while strains that do not produce curli remain white [41]. The Keio collection consists of 3985 nonessential gene mutants comprising more than 90% of the 4390 open reading frames of *E. coli* K-12 (named wild type, WT, in the remaining manuscript) [40]. Following growth in conditions that favor curli production (26 °C for 48 h), strains with changes in CR phenotypes were isolated and scored according to their color, which varied from the white color of *csgA* to the darker red of *cpxR* (Figure 1B). Genes known to affect curli production, such as curli specific genes (*csgABEFG*) and previously described transcription factors (*cpxR* and *ompF*) were identified, validating our screening technique. Strains such as *thyA*, *glnA*, *ubiE* and *ubiF*, did not grow well under the conditions tested and were not further analyzed (see *ubiF* Figure 1A and Table S1 strains marked with ‘).

Strains with an altered phenotype were regrown on CR indicator plates for verification and scored numerically according to color from lightest to darkest (Figure 1B and Table S1). We isolated 332 Keio mutants with reproducible CR phenotypes. Of these, 64 strains had two or more phenotypes upon isolation of single colonies. Each genotype from these gene deletion strains was verified by polymerase chain reaction (PCR), revealing that most strains had the correct Kan^r^ insert for each phenotype (Table S2). We excluded the 64 mixed phenotype strains from further study as they likely have second site suppressors. However, we included the strains *cmk*, *fabF*, *mdoG*, *pgm*, *trpD*, *trpE*, *yiaK*, and *ymgE* because both of their CR phenotypes were defective (lighter red than WT cells). The resulting 276 mutants were 14.3% white, 16.5% light pink, 27.2% pink, 18.6% light red, and 23.3% dark red when grown on CR indicator plates (Figure 1C). To better quantify curli production in the identified mutants, CsgG and CsgA protein levels were measured in whole cell extracts by Western blotting (sample of color groups Figure 1D and Table S1). Protein levels were generally consistent with CR phenotypes (Figure 1D). In some light pink mutants, the curli secretion protein CsgG [23,24,25] was present; however, no CsgA was detectable (Table S1).

**Figure 2:**
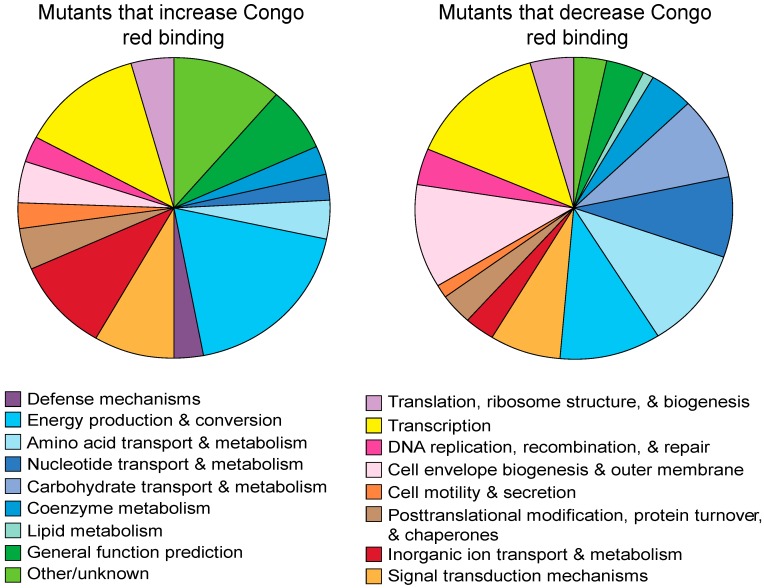
Genes affecting curli production perform diverse roles. Genes identified for altered curli production sorted by clusters of orthologous groups (COGs) showed involvement of regulation, environmental sensing, metabolism, cell envelope biogenesis, transport, and protein turnover in curli production.

We divided the gene mutations with altered CR binding into clusters of orthologous groups (COGs) [40,42,43,44,45] and used Echobase [46] to assign a cellular location to each protein product (Table 1 and Table S3, Figure 2). Collectively, the genes identified in the CR screen are predicted to encode proteins that are primarily localized to the cytoplasm. Genes in the transcription COG (K) are likely to have collateral effects as regulators often control multiple gene products, such as the case with cAMP receptor protein (CRP) which has been shown to have 70 unique targets [47]. Because of this, of the 43 transcription related genes, only those with previously reported relationships or verified binding sites on the *csg* intragenic region were studied. The CR phenotypes of the Keio collection strains were mostly consistent with the literature: exceptions were found in cyclic-di-guanosine monophosphate (c-di-GMP) and regulatory proteins, some of which are known to have strain-dependent effects on curli production [48] (Table S4). In the following sections, we explore genes from several different functional groups and discuss their likely roles in curli production.

### 2.2. Cell Envelope

**Figure 3:**
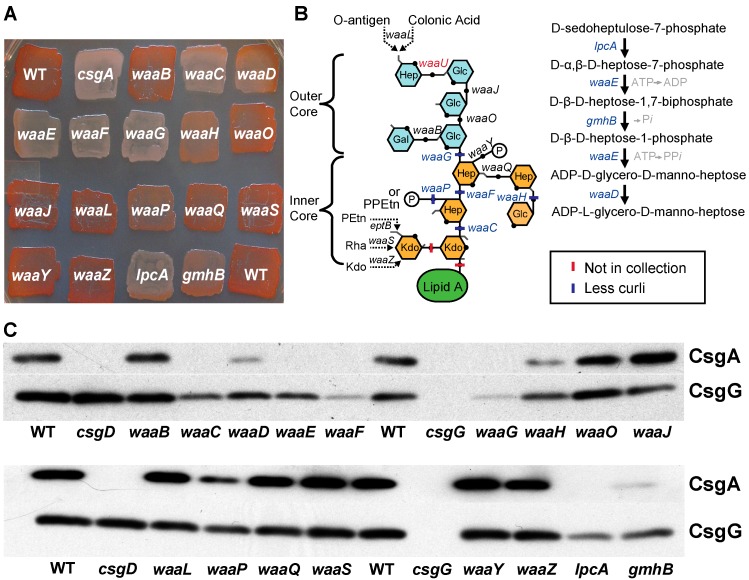
Lipopolysaccharide (LPS) mutants are defective for curli production. (**A**) LPS mutant strains and BW25113 (WT) grown on CR indicator plates at 26 °C for two days. (**B**) A schematic of LPS structure. Blue lines represent curli defective LPS mutants. Red lines represent LPS genes not in the Keio collection. (**C**) Whole cell Western blots of LPS mutants probed with anti-CsgG and anti-CsgA antibodies. All samples were grown on YESCA plates at 26 °C for two days and treated with formic acid.

Curli assembly occurs at the cell surface, therefore, it is not surprising that genes involved in cell envelope and outer membrane biogenesis have defects in curli assembly (COG family group M) [49]. The cell envelope includes the inner membrane, the periplasm, the outer membrane, and extracellular structures including fimbriae and extracellular polysaccharides (EPS) [50]. Multiple lipopolysaccharide (LPS) biosynthesis gene mutants, especially those involved in assembling the inner core region of LPS, were defective for curli production (Figure 3A,B). These genes encode hexose and heptose transferases, enzymes that produce the inner core sugar building block adenosine diphosphate (ADP)-l-glycero-d-manno-heptose, the LPS kinase *waaP*, and the transcriptional antiterminator for the *waaQGPSBIJYZK* operon *waaH* [51,52,53,54]. Western blot analysis showed that many LPS mutants with CR defects had corresponding decreases in CsgA and CsgG levels (Figure 3A,C compare WT to *lpcA*, *gmhB*, and *waa* mutants). Mutants in the inner core sugar transferases *waaC*, *waaF*, *waaG*, and the heptose biosynthesis genes *lpcA* and *waaE* were the most defective in CR binding and these strains had lower CsgG levels compared to WT and no CsgA was detected (Figure 3C). 

Previous studies have implicated the LPS biosynthesis genes *waaG, ddhC (rfbH)*, and *lpxM* (*msbB*) as being important for curli production [55,56]. In *Salmonella enterica*, both *waaG* and *ddhC* strains produce less curli than WT [55]. Similarly, the *waaG* strain in K-12 *E. coli* has reduced curli production. [57,58]. We also looked at other lipid A modifying enzymes to see if they affected curli production. The *lpxL*, *lpxM* (*msbB)*, *lpxP*, *pagP*, and *arnT* mutant strains all produced curli at WT levels suggesting the modification state of the lipid A core does not necessarily affect curli production in K-12 strains of *E. coli* (Figure S2C, left plate). 

**Figure 4:**
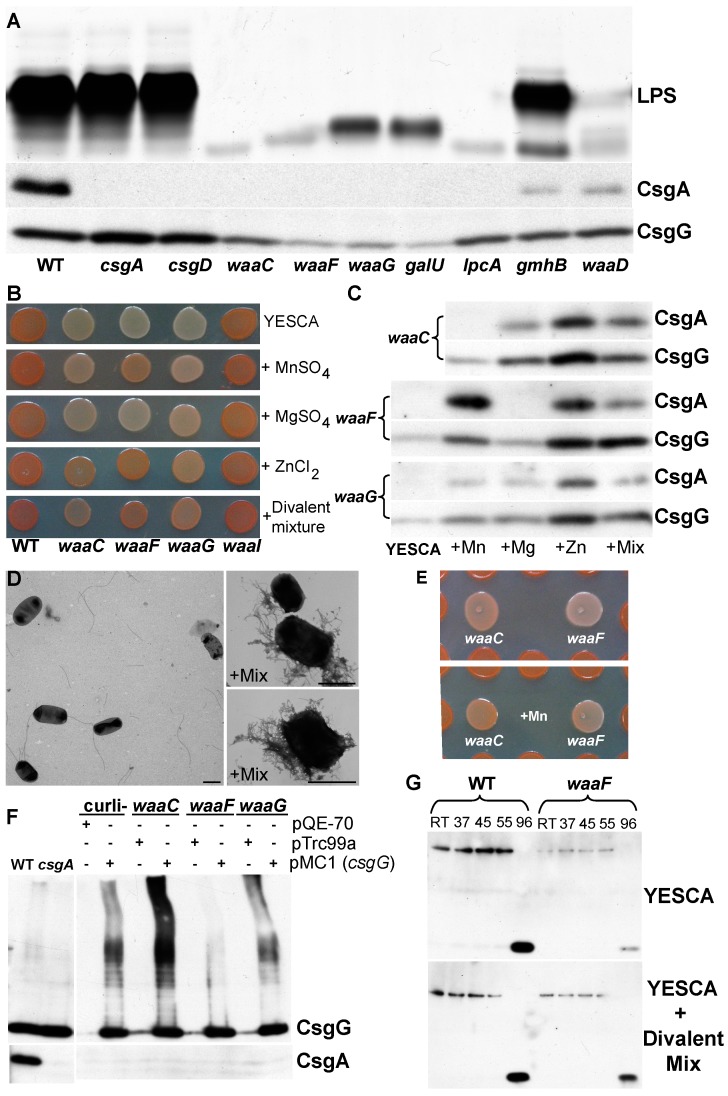
Effects of inner core LPS mutants on curli production. (**A**) Silver stain of LPS from LPS mutants and BW25113 (WT) resolved on a 14% polyacrylamide gel electrophoresis (PAGE) Tricine gel (top panel), and the respective whole cell Western blots probed with antibodies to CsgA and CsgG are shown in the bottom part of panel A. (**B**) Addition of divalent salts MnSO_4_ (0.5 mM), MgSO_4_ (0.5 mM), ZnCl_2_ (0.1 mM), or a mixture of all three divalent and CaCl_2_ (0.5 mM) to CR indicator plates had varying abilities to suppress the CR phenotype of the indicated LPS mutants. (**C**) Whole cell Western blots of LPS mutants probed with anti-CsgG and anti-CsgA antibodies. All samples were grown on YESCA plates supplemented with the indicated salts at 26 °C for two days and treated with hexafluoroisopropanol (HFIP). (**D**) Transmission electron microscopy (TEM) images of *waaF* grown on YESCA and YESCA amended with a mixture of divalent cations. Scale bar equals 1 µM. (**E**) The addition of 2 μL of 0.1 M MnSO_4_ to LPS mutants *waaC* and *waaF* resulted in their ability to bind CR only when they were grown on plates surrounded by BW25113. (**F**) Overexpression of *csgG* from pMC1 did not rescue CsgA secretion in inner core LPS mutants. (**G**) The CsgG of WT (BW25113) and *waaC* had similar mobility in a 13% sodium dodecyl sulfate (SDS)-PAGE gel after a 10 min treatment at the indicated temperatures as in previous studies [26] except samples were 10 μL of 0.5 OD_600_ resuspended cells grown on YESCA for 2 days at 26 °C. The Divalent mix was MnSO_4_ (0.5 mM), MgSO_4_ (0.5 mM), ZnCl_2_ (0.1 mM), and CaCl_2_ (0.5 mM).

Analysis of mutations in other LPS synthesis genes revealed that curli production is likely affected by genes that synthesize or transfer the ADP-l-glycero-d-manno-heptose to Lipid A-KDOII [52] (Figure 3A,B). Mutations in *waaD* lead to an accumulation of heptose free LPS [59], and *waaD* mutants had decreased curli production compared to WT (Figure 3C and Figure 4A). Mutations in *gmhB* also accumulate heptose-free LPS and, like the *waaD* mutants, the *gmhB* strain had decreased curli production (Figure 3A) [60]. The composition of the LPS in the *waaD* and *gmhB* strains may explain their intermediate curli phenotype. Unlike the *waaG* mutant strain and other inner core mutants, both *waaD* and *gmhB* strains have a small amount of glucose I and further modified LPS [60,61] (Figure 4A). Accordingly, a mutation in *waaI*, which has fully glucose I modified LPS [52], is unaffected for curli production. Similarly, *waaP*, which lacks phosphorylation of heptose I [52], had less curli production and partially substituted LPS (Figure 4A). Thus, it appears that even a small amount of glucose I modified LPS is capable of supporting curli subunit secretion and assembly into an amyloid fiber. 

Several LPS mutant strains had a notably dry and crumbly texture when dragged across the plate (Figure S1A) [17,53,62], including *galU* and *waaG* and to a lesser degree *waaF*. GalU is needed for growth on galactose and trehalose and is required for the production of uridine diphosphate (UDP)-d-glucose, trehalose, the LPS outer core, colonic acid, and periplasmic glucans [63,64,65,66]. Since WaaG adds UDP-d-glucose to the inner core of LPS, the *galU* and *waaG* mutants should be functionally similar. Both strains displayed a light pink phenotype and had similarly low levels of CsgG by Western blotting. These mutants were also visibly drier than WT, crumbled when scraped from plates [62], autoagglutinated when resuspended in PBS [53], and displayed identical LPS profiles on silver stained polyacrylamide gel electrophoresis (PAGE) gels [66] (Table S1, Figure 4A, Figure S1 compare *galU* and *waaG*). Because of the dry colony phenotype of the *waaG* and *galU* strains (Figure S1A), we tested the cell hydrophobicity of the inner core sugar transferase mutants using the bacteria adhesion to hydrocarbons (BATH) method [67]. Mutant strains that had less developed LPS cores were more hydrophilic as measured using the BATH method (compare *waaC*,*F*,*G* in Figure S1B and Figure 4A). As expected, both *galU* and *waaG* had similar cell hydrophobicity profiles. Because of the significant changes made to the membrane in the *galU* and *waaG* mutants, we looked at the integrity of the CsgG protein present within the membrane.

Because CsgG is required for the stability and secretion of CsgA and CsgB [23,24,25], and several of the inner core LPS mutants had decreased levels of CsgG protein (Figure 4A), we asked if ectopic overexpression of CsgG could rescue the curli assembly defect in the inner core LPS mutants. Expression of *csgG* from plasmid pMC1 increased the amount of CsgG protein in the *waaC*, *waaF* and *waaG* strains, but was unable to restore CsgA protein levels or curli production (Figure 4F). Interestingly, the transcript levels of *csgD*, *csgA*, and *rpoS* were near WT levels in the *waaF* strain (Figure S3). Taken together, these data suggested that the CsgG present in inner core LPS mutants was not able to properly fold or function in the outer membrane. To test this idea, we supplemented CR indicator plates with divalent cations. Divalent cations interact with LPS molecules in the outer membrane (OM) [52,68], and their presence can stabilize outer membrane porins [69,70]. When added to CR indicator plates, the divalents Mn^2+^, Mg^2+^, and Zn^2+^ all increased CsgG levels and partially complemented CsgA secretion and curli production in inner core LPS mutants (Figure 4B–D). Addition of Mn^2+^ near inner core mutants also resulted in increased CR binding (Figure 4E). Many LPS mutants displayed an aberrant CR phenotype: a rim of darker stained cells near the edge of the plate (Figure 3A and Figure S1A) where there is less competition for limiting divalent cations. The mechanism of complementation by addition of divalent cations is unclear. The divalent cations may be interacting with phosporhyl moieties to reshape the OM and decrease the higher phospholipid content of the OM outer leaflet in inner core LPS mutants [68]. Alternatively, they may stabilize the interactions between LPS and CsgG or a partner OM protein; however, addition of divalent cations did not alter the heat modified mobility of CsgG from *waaF* relative to WT (Figure 4G), suggesting divalent cation rescue of CsgA levels is not due to direct interaction with CsgG. 

Other components of the cell envelope, including the enteric common antigen (ECA) and the periplasmic glucans, were found to affect curli production (Figure S4). Mutants in ECA biosynthesis with altered curli production include *rffA*, *rffC*, *rffT*, *wzxE*, and *rfe* (Figure S4B), which are involved in synthesis or addition of thymidine diphosphate 4*N*-acetyl-α-d-fucosamine (TDP-Fuc4NAc) to lipid II (Figure S4A) [71] and have increased DegP levels via σ^E^ and Cpx induction [72]. These results suggest ECA was not required for curli production, as only mutants accumulating lipids II and III were curli deficient. Conversely, the *rfe* strain, which accumulates undecaprenyl-P, produces more curli than WT cells (Figure S4B). Mutants in *rfe* suppress the activation of *degP* in the *rffA*, *rffH*, and *rffT* strains suggesting undecaprenyl-P and lipid II accumulation have opposing roles in envelope stress responses and curli production [72]. The opposing curli phenotypes within ECA mutants are consistent with previous studies that demonstrated that increased σ^E^ activity has a negative effect on curli production [73]. ECA mutants that induce the σ^E^ or Cpx stress response make less curli than WT, while strains with lower induction produce more curli (Figure S4B). Similarly, mutations in *tolA* and *pal* have higher σ^E^ levels [74] and were defective for curli (Table S1). The *rseA* strain*,* which lacks the anti-sigma factor of σ^E^ [75,76,77], produced less curli and was light pink on CR indicator plates (Figure S4C). Furthermore, ectopic expression of the anti-sigma factor *rseA* in WT resulted in increased curli production (Figure S4D). The σ^E^ stress response may function to limit the production of extracellular fibers during outer membrane stress. However, the *csg* genes lack the σ^E^ consensus sequence and overproduction of σ^E^ did not significantly affect their transcript levels [78,79]. 

Defects in LPS biosynthesis also result in σ^E^ induction [80,81]. Inner core LPS mutants such as *waaC*, *waaD*, and *galU* have drastically altered outer membrane protein profiles [53,62], increased σ^E^-dependent transcription [82,83,84], and produce little or no curli (Figure 3C). The Cpx two-component system, which also negatively regulates curli specific genes [85,86], is induced in many of these LPS mutants [72,84]. Furthermore, Zn^2+^ rescued curli production in some inner core LPS mutants (Figure 4B,C), and has been shown to induce σ^E^ and σ^E^-regulated genes [87,88]. The expression of *rseA* or *rseAB* in the *waaC*, *waaF*, or *waaG* mutants [77,89] could not complement curli production. Thus, while σ^E^ and Cpx have a role in modulating curli production, it is more likely that inner core mutants produce less curli due to a secretion defect in CsgG.

### 2.3. Carbohydrate Metabolism, Energy Production, and Gluconeogenesis

Strains with mutations in several genes involved in global carbohydrate flux and sugar import were found to be defective for curli production: *cyaA*, *crp*, *fruR* (*cra*), *ptsH*, *ptsI*, *aceE*, *fbp*, *gnd*, *tktA*, *tpiA* (Table S1)*.* The metabolic flux changes of the Keio *cyaA*, *crp*, and *fruR* strains have been examined under different growth conditions and shown to produce less phosphoenolpyruvate (PEP) from oxaloacetate in glucose limiting conditions [90,91]. PEP is used by the phosphotransferase system (PTS) to transport and phosphorylate many different sugars. FruR increases PEP production from pyruvate through a combination of *pykF* repression and *ppsA* activation [92]. The cAMP–CRP complex also activates *ptsHI-crr* [93], and *cyaA* and *crp* mutants, both of which are defective in cAMP–CRP mediated gene activation, and have low levels of glucose uptake due to a PTS defect [94]. Combined with the curli phenotype for the PTS genes, *ptsH* (Enzyme I) and *ptsI* (HPr), these results suggest that a defect in the PTS system leads to lower curli production. However, many of these gene products have global effects on gene transcription. The cAMP–CRP complex regulates multiple genes including direct activation of *csgDEFG* [93,95]. 

Enzymes for central metabolism, energy production, and their coenzymes also play an important role in curli production. Citric acid cycle (TCA) mutants with reduced curli production include genes encoding enzymes for the complete conversion of α-ketoglutarate to fumarate: *sucA*,*B*,*C*,*D* and *sdhA*,*B* (Tables S1 and S5). The restriction of curli defective mutants to the upper TCA cycle and the curli defects in *fruR*, *fbp*, *tpiA*, and *sfcA* (*maeA*) indicate that gluconeogenesis is required for curli production. FruR tightly regulates gluconeogenesis by increasing gluconeogenic enzymes and decreasing glycolytic enzymes to prevent a futile cycle [96]. In *S. typhimurium*, a curli producing WT strain had higher levels of gluconeogenic end products including glucose, glycogen, and trehalose as well as lower levels of succinate, fumarate, malate, and polyamines relative to a *csgD* strain (Table S5) [97]. Gluconeogenic-specific genes including *pckA*, *maeB*, *ppsA*, and *fbp* were also upregulated in the WT strain, and a *ppsA pckA* double mutant in *S. typhimurium* was defective for curli and glycogen production [97]. Thus, gluconeognesis appears to not only be coregulated with but also required for curli production.

Gluconeogenic metabolism is important for pathogenesis in uropathogenic *E. coli* (UPEC) [98]. Like urine, the media used to express curli including YESCA and colonization factor antigen (CFA) are mostly composed of amino acids and small peptides. When used as a carbon source, many amino acids are broken down into pyruvate or compounds of the upper TCA cycle such as α-ketoglutarate, succinate, and formate. Glucose produced by gluconeogenesis is used in LPS, glycogen, trehalose, osmoregulated periplasmic glucans (OPG), or various EPS including cellulose, ECA, and colonic acid. Mutants in most of the pathways utilizing glucose were not defective for curli production. Interestingly, outer core LPS biosynthesis requires UDP-d-glucose which is converted from glucose-6P through the action of Pgm and GalU (Table S1) and added to the heptose II of inner LPS core by WaaG [52]. The *waaG*, *pgm*, and *galU* strains were all defective for curli production, suggesting gluconeogenic mutants may lack curli due to glucose I defective LPS. Mutants that lack glucose-I LPS modifications can be partially suppressed by the addition of divalent ions (Figure 4B). Indeed, the addition of divalent ions to *sdhA* and *sdhB* resulted in increased CR binding, suggesting that the lack of the glucose-I LPS modification is at least partially responsible for the curli defect in gluconeogenesis mutants (Figure S4E).

Anaerobic respiration is vital for *E. coli* to persist as a pathogen [99]. *oprF* mutants in *Pseudomonas aeruginosa* have impared anaerobic respiration and as a result, have significantly reduced anaerobic biofilm production [100]. Several terminal dehydrogenase components (*sdhA–D*) produced altered CR binding phenotypes (Table S1), where loss of the catalytic domains, *sdhAB,* lead to lower CsgA levels and the loss of stabilizing domains, *sdhCD*, lead to higher CsgA levels. Additionally, *narQ*, the primary sensor of the presence of nitrate, has been shown to alter biofilm formation and motility [101,102], and in our study yielded decreased CsgA and CsgD levels (Table S1). Taken together, these respiration mutants point to the involvement of the electron transport chain in curli production.

Previously, our lab investigated the effect of *nagA* mutants on curli production [35]. In the Keio collection, we found *nagA*, *nagC*, and *nagK* mutants to be defective for curli production (Table S1). For the *nagA* strain, the decrease in curli production was similar to other K-12 strains, and less than seen in the C600 strain [35]. Intriguingly, we found *yhbJ* and *pcnB* strains were dark red on CR plates and produced significantly more curli than the WT strain. YhbJ has recently been shown to destabilize the RNA *glmZ*, which increases the *glmS* transcript stability [103,104]. *glmZ* also regulates curli production independent of CsgD protein levels [105]. The *glmS* transcript can be polyadenylated by PcnB and rapidly degraded [106]. GlmS transfers ammonia to fructose-6-P to form GlcN-6-P, which is later converted to UDP-GlcNac [64]. Fructose-6-P is the product of NagA degradation of GlcNac-6-P [35], suggesting that the curli defect in a *nagA* strain may also be due to lower amounts of fructose-6-P. The resulting lower UDP-GlcNac levels, which are needed for lipid A, ECA, and peptidoglycan biosynthesis, may lead to a compromised cell envelope and subsequently to lower curli expression. The curli defect of the Keio *glmM* mutant would seem to confirm this; however, *glmM* has been reported to be an essential gene [107], and therefore its presence in the Keio collection suggests that the *glmM* strain used in our study has acquired suppressor mutations (Table S1). We also found decreased curli in *nanK* and *nanE* (Table S1). Both are involved in sialic acid biosynthesis, which ultimately is converted to GlcNAc-6P [64] supporting the role of fructose-6-P in minimal cell envelope development for curli production. 

### 2.4. Multiple Regulatory Networks Control Curli Gene Expression

At 754 bases, the non-coding region between *csgD* and *csgB* is the fifth largest region between divergent operons in *E. coli* K-12 and the thirteenth largest intergenic region overall (Table S6 and Figure S5). The intergenic region has strong inherent curvature and an AT content of 65.5% [108], which aids binding of nucleoid proteins such as IHF and H-NS which can induce sharp bending in DNA [34]. At least twelve proteins and five small RNAs have been shown to bind within the intergenic region between *csgD* and *csgB* [34,64,109,110] (Figure S5). Previously, most genes found affecting curli production did so through transcriptional changes at one or both curli operons [16] or by modifying cyclic-di-GMP metabolism [111] (Table S4). We identified many additional regulatory elements that also affect curli production. In fact, more than a fifth of the genes hit in the CR screen encode either signal transduction proteins or transcription factors (Table 1). The high number of regulatory proteins and the large intergenic region between *csg* operons, are consistent with curli biogenesis being an intricately-regulated process. Indeed, 26 of the 32 mapped protein binding sites are within 200 bp of the transcription start of *csgDEFG* operon, the highest such density in *E. coli* (Figure S5 and Table S6). 

Several mutants identified in our screen have been shown to directly or indirectly regulate σ^S^ levels or function, including *crp*, *clpP*, *clpX*, *dksA*, *dnaK*, *galU*, *hns*, *hfq*, *nlpD*, *nuoG*, *pgm*, and *mdoA (mdoGH)* [112,113,114,115]*.* Similar to an *rpoS* mutant, an *nlpD* mutant completely lacks curli, probably due to loss of the major *rpoS* promoter within *nlpD* [116]. Since DksA affects ppGpp-dependent induction of σ^S^ [117], we tested whether altering ppGpp production affected curli production. An MG1655 *relA spoT* double mutant, which is defective in ppGpp synthesis [118], was light pink on CR plates and produced almost no curli proteins (Figure S6A). When the same mutants were made in the BW25113 strain, the CR binding phenotype was more severe and no CsgD or curli were detected by western blotting (Figure S6B). In addition to *rpoS*, we found that deletion of the sigma factor genes *rpoN* and *rpoZ* decreased curli production (Table S1), underscoring the complexity of curli regulation by different sigma factors.

Several genes involved in quorum sensing and virulence were found to affect curli production including *qseB*, *qseC*, *aaeR*, *lsrF*, *ygiU* (*mqsR*), *sdiA*, and *flgM*. The *qseC* strain produced more curli than WT; however, *qseB* had mostly light pink colonies with a few dark red suppressor colonies (Tables S1 and S4). Previously, QseC but not QseB was found to be important for curli, type I pili, and flagella production in uropathogenic *E. coli* [119]. The Keio *flgM* strain overproduced flagella and made less curli consistent with the antagonistic relationship between these two extracellular appendages [109,120]. Similarly, the *hdfR* strain, which lacks a repressor of the *flhCD* operon, was defective for curli. However, *sdiA*, which also overproduces flagella [121], had higher levels of curli production (Table S1).

**Figure 5:**
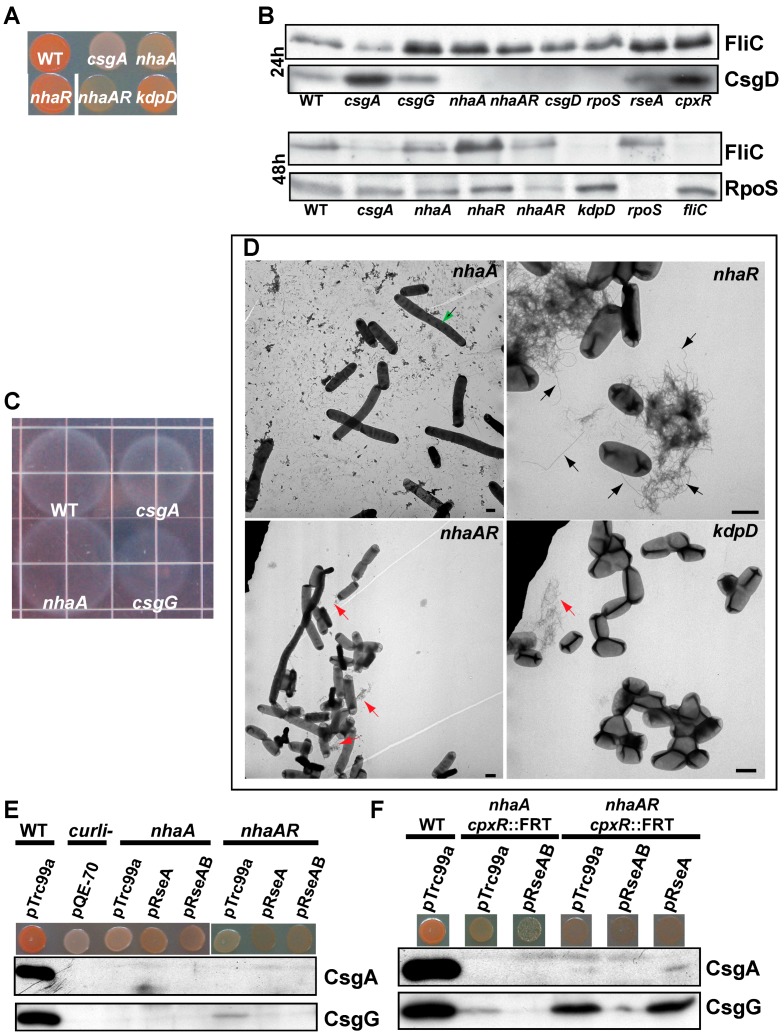
Sodium antiporter gene *nhaA* is required for curli production. (**A**) Strains were grown on CR indicator plates at 26 °C for two days. (**B**) Whole cell Western blots probed with antibodies to CsgA, CsgG, and FliC at 24 and 48 h. All samples were treated with HFIP; (**C**) Motility of WT, *csgA*, *csgG*, and *nhaA* strains in 0.2% YESCA motility plates at 26 °C. (**D**) TEM images from cells grown for 26 °C for two days on YESCA plates. Black arrows indicate flagella. Red arrows indicate curli. Green arrows indicate filamentous cells. Scale bar equals 1 µm. (**E**) Expression of *rseA* in trans using pRseA or pRseAB does not rescue curli expression in *nhaA* or *nhaAR* strains as detected by CR binding or Western blot probed with antibodies to CsgA or CsgG. (**F**) Expression of *rseA* in trans from pRseA or pRseAB does not rescue curli production in *nhaA cpxR* or *nhaAR cpxR* double deletions as detected by CR binding or Western blot probed with antibodies to CsgA or CsgG.

A mutation in the Na^+^:H antiporter encoding gene *nhaA* resulted in cells that stained light pink on CR indicator plates, and had much lower levels of CsgA and CsgG (Figure 5A,B,D and Table S1). NhaA is one of three sodium ion antiporters which uses the proton electrochemical gradient to expel sodium ions [64], and had been loosely linked to amyloid production in *Shewanella* [122]. The *nhaA* mutant was more motile than WT (Figure 5C), had more FliC protein when measured by western blot (Figure 5B), and more flagella than wild type when examined by transmission electron microscopy (TEM) (Figure 5D). When bound to Na^+^, NhaR activates *nhaA* and other genes including *pgaABCD* and *osmC* [64]. Since high levels of intracellular Na^+^ might result in a constitutively active NhaR, we made a *nhaAR* deletion, which did produce a small amount of curli (Figure 5D).

Curli fibers are maximally produced at room temperature in low salt conditions [19]. High salt and osmolarity typically repress *csg* transcription through the OmpR/EnvZ and Cpx systems [86], which respond to high osmolarity in the periplasm. However, YESCA is a low salt medium able to support curli production in *E. coli*. High Na^+^ also inhibits the proper assembly of FtsZ in vitro [123]. When we examined *nhaA* and *nhaAR,* we found many filamentous cells (Figure 5D). Similary, a *kdpD* mutant displayed cell division defects and had reduced curli production (Figure 5B,D). KdpD regulates the influx of potassium, which promotes FtsZ assembly [64]. Because high levels of CpxR-P results in aberrant cell division [124], we examined the levels of Cpx and σ^E^ regulated genes in *nhaA* and *nhaAR.* Double deletions of *nhaA cpxR* and *nhaAR cpxR* both produced more curli than *nhaA* and *nhaAR* (Figure 5F); however, over expression of *rseA* or *rseAB* resulted in lower curli production (Figure 5E). Collectively, these results suggest the high intracellular sodium in *nhaA* strains is inhibiting cell division and results in Cpx induction which decreases curli production.

**Figure 6:**
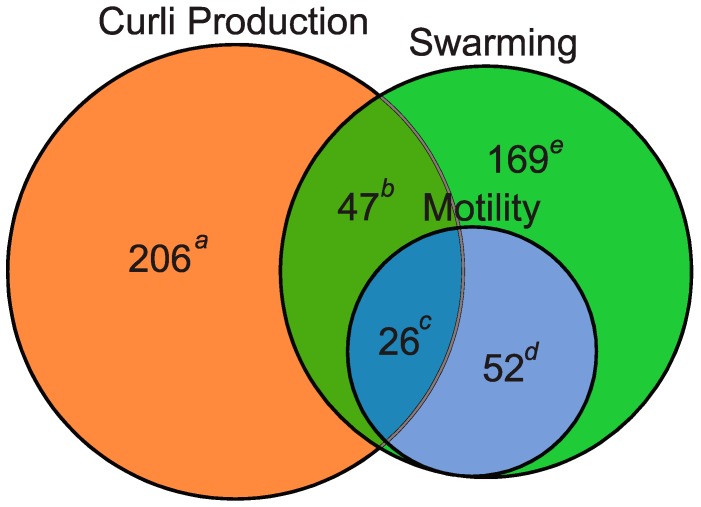
Venn diagrams demonstrating few genes associated with curli affect swarming or swimming. Comparison of genes found to affect curli with swimming and swarming associated genes. Sections of Venn diagrams: ^a^ (orange), curli associated genes that do not affect swarming or swimming motility; ^b^ (dark green), curli associated genes that affect swarming motility; ^c^ (dark blue), curli associated genes that affect swimming motility; ^d^ (light blue), swimming defective genes that do not affect curli; and ^e^ (light green), swarming defective genes that do not affect curli. See Table S7 for complete list of genes.

The master biofilm regulator, CsgD, has been shown to affect the transcription of genes outside the curli operon, including those promoting biofilm production, gluconeogenic metabolism, and peptide import (see Table S5). CsgD also plays an important role in decreasing flagella rotation and production, promoting the switch from single planktonic growth to community behavior, through the activity of AdrA, ci-di-GMP, and σ^S^ as well as direct repression of the *fliE* and *fliFGHIJK* operons [109,120]. Here, *flgM, flhC, fliI, fliG,* and *fliT* had altered curli production (Tables S1 and S4). To further explore the intersection of flagella and curli we compared the results from our screen on CR indicator plates with other screens involving the Keio collection. Inoue et al. [125] screened the Keio collection for defects in swarming motility using Eiken Agar and subsequently checked the swarming mutants for reduced swimming motility [125,126]. Using GeneVenn [127], motility genes identified by Inoue et al. were compared to the genes identified in our screen (see Figure 6). Very few genes were found to overlap, especially between swimming motility and curli production. Half the genes that affect both swimming motility and curli encode for either adenosine triphosphate (ATP) synthase or LPS biosynthesis genes (Table S7). A second study looked for biofilm defective mutants in the Keio collection using crystal violet and 96-well plates [128]. The biofilm mutants were subsequently tested for flagella, type I pili, and curli production [129]. For curli production, growth was for three days on CFA amended with CR but not Coomassie brilliant blue. Comparison of flagella, curli, and type I pili genes also identified little overlap between flagella and curli associated genes (Table S7). However, fewer curli genes were identified, perhaps due to the use of LB (Luria-Bertani) media in the initial biofilm screen [129]. LB media has relatively high salt concentration, which inhibits curli production [19]. Additionally, the CR phenotypes presented here are different for several strains listed in the LB screen; for example, several inner core LPS mutants are listed as WT for CR binding in their study [129]. Consequently, we tested our strains using similar conditions and found that several of the phenotypic differences were due to media, growth, or staining differences (Compare CR plates in Figure S2, Table S8).

### 2.5. CsgD Transcript and Protein Levels Are Altered in Several Strains with CR Phenotypes

CsgD is considered the “master” biofilm transcription factor [19,130]. We selected a total of 38 mutant strains, sampling each CR phenotype and representing each COG, to further investigate the regulation of *csgD* at both transcript and protein level.

**Figure 7:**
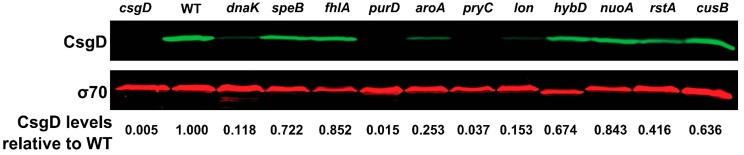
CsgD protein levels are changed in several mutants. A representative western blot is shown for the data presented in Table S9. CsgD protein levels are shown in green and σ70 levels are shown in red as a loading control. CsgD levels were normalized to σ70, and then each mutant was normalized to WT to yield the relative CsgD protein levels for each mutant list of genes. σ70 migrates father in the *hybD* line consistently over multiple trials on different days.

*csgD* transcript levels were measured in all 38 strains using quantitative real time polymerase chain reaction (RT-qPCR) (Table 2 and Table S9) and CsgD protein levels were measured by Western blot analysis (Table S9 and representative blot Figure 7). Twenty-one of the 38 strains had *csgD* transcript levels that were significantly different from WT (Table 3). There was a correlation of CR phenotypes with *csgD* transcript and CsgD protein levels. For example, of twelve mutants tested that had increased CR binding, eight had increased levels of *csgD* transcripts and ten had increased levels of CspD protein (Table 2 and Table S9). Mutants *mdoC*, *perR*, and *cusB*, which presented increased *csgD* transcripts, did not have a detectable change in CsgD protein levels while *truB* and *qseC* exhibited no change in *csgD* transcripts to account for the increase of CsgD levels (Table 2 and Table S9). Of 19 mutants that were white, light pink, or pink on CR plates, 57% of them also had significantly decreased *csgD* transcript levels (Table 2) and all but *nagA*, *dksA*, *aaeR*, *glvG*, *cmr*, and *php* had decreased CgsD levels consistent with their CR coloring and qPCR data (Table S9). There were also notable cases where the *csgD* transcript or protein levels did not predictably correlate with CR binding. 

Two mutants that were pink on CR plates, *nagA* and *dksA*, had significantly increased *csgD* transcript levels (Table 2). However, it appears that the 16s ribosomal RNA (rRNA) levels were changed in these mutants and when we normalized by total RNA, the c*sgD* transcript levels were similar to WT (Table 4). It is also worth noting that a threefold increase in *csgD* transcripts was observed in *cysB*, but CsgD protein levels were half of WT, whereas *fes* was observed to have *csgD* transcript levels nearly identical to wild-type with approximately half the levels of CsgD protein (Table S9).

The *csgD* transcript data presented here agree with some previously published findings. For instance, mutants in pyrimidine synthesis, *pyrC*, were shown to have decreased *csgD* transcripts [131] and we observed similar decreases in *csgD* expression in a *purD* mutant (Table 2). It is interesting to speculate that these two mutants act through the same mechanism, but more work is needed to elucidate how *pyrC* and *purD* contribute to curli biogenesis. Hfq, a chaperone protein responsible for RNA stability, is required for *csgD* expression in *S. enterica* [132], and we confirmed that this is true in *E. coli* (Table 2). Additionally, RcsB represses the *flhDC* operon [133], induces expression of the small RNA (sRNA) *rprA* [134]*.* In turn, the sRNA *rprA* can reduce *csgD* transcription and CsgD protein levels [134]. We observed that an *rcsB* mutant strain had increased *csgD* transcripts and CsgD protein levels (Table S9). Taken together, these data suggest that the increased CR binding observed in *rcsB* mutants is likely the result of lower RprA levels. Two other sRNAs, McaS and GcvB, have also been shown to target *csgD* transcripts [135]. McaS has been found to interact with Hfq implicating additional levels of sRNA control on *csgD* [136]. 

The CR screen also revealed possible post-translational control of CsgD (Table S9). For example, *hdfR*, *mltA*, *and fabH* mutant strains had normal *csgD* transcripts, but decreased CsgD protein levels, suggesting that these genes are required for the proper translation of *csgD* or CsgD protein stability (Figure S3 and Table S1). Consistent with this, *hdfR* and *mltA* have been shown to interact with the *csgD* messenger RNA (mRNA) [137]. The FabH protein is an initiator of fatty acid synthesis capable of conjugating acetyl-CoA to acyl-carrier protein (ACP), and lack of *fabH* or overexpression leads to decreased fatty acid chain length and abundance [138,139,140]. The mechanism behind how FabH changes CsgD protein levels warrants further investigation. 

An intriguing result from the screen was that there were multiple mutants, *ddpD*, *glvG*, and *php*, which had altered CR binding phenotypes, yet normal *csgD* transcript and CsgD protein levels (Table S9). For example, the *php* mutant was white on CR plates, had undetectable levels of CsgA and CsgG, but had WT levels of CsgD protein (Tables S1 and S9). Taken together, we suggest that CsgD is inactive in these mutant strains, or that CsgBA cannot be produced or appropriately secreted because CsgG is not present. In any case, it is clear that *php* is essential for curli biogenesis and it acts downstream of CsgD transcription and translation. 

## 3. Materials and Methods

### 3.1. Bacterial Strains and Growth Conditions

The Keio collection [40] was made by the Datsenko and Wanner method in *E. coli* strain BW25113 [141]. The collection was shipped to us after being grown on LB. Freezer stocks made with LB broth (10 g tryptone, 5 g yeast extract, 10 g NaCl) with 20% glycerol were maintained in 96-well microplates at −80 °C. Additional strains and plasmids used are listed in Table S10. A complete curli deletion strain was made in BW25113 so that both the *csgDEFG* and *csgBAC* operons were deleted using *csgG* and *csgC* terminal primers [40] the method of Datsenko and Wanner, 2000 [141].

Typically, bacteria were grown for 48 h at 26 °C on YESCA plates (1 g yeast extract, 10 g casamino acids, and 20 g agar per liter). Congo red indicator plates are YESCA media amended with 50 μg/mL CR and 10 μg/mL Coomassie Brilliant Blue (CBB). CFA agar (1.5 g yeast extract, 10 g Casamino Acids, and 20 g agar per liter containing 0.4 mM MgSO_4_ and 0.04 mM MnCl_2_ buffered to pH 7.4) with 100 μg/mL CR or 50 μg/mL CR and 10 μg/mL CBB were sometimes used. For the experiments looking at the effect of divalent cations, salts were added to CR indicator media prior to autoclaving. To remove residual salts in these experiments, strains were grown to saturation in LB overnight, washed and diluted five-fold in YESCA, and spotted onto the appropriate plates. LPS mutants were always surrounded by other strains due to differences in CR binding near the colony edge. Antibiotics were added when appropriate in the following final concentrations: 25 µg/mL kanamycin; 25 µg/mL chloramphenicol; or 100 µg/mL ampicillin.

To screen the Keio collection, we used sterile toothpicks and plate bolt replicators to copy the collection onto CR indicator plates amended with 25 µg/mL kanamycin. Following growth for 48 h at 26 °C, the strains were scored for color as indicated. If a colony was pink or darker red than the surrounding strains it was restreaked for single colonies. The CR phenotype of each strain was verified by comparison to BW25113. To emphasize CR phenotypes in the pictures captured, the levels were uniformly adjusted by setting the gray point to a clear spot on the red agar in Adobe Photoshop.

### 3.2. Western Blotting and LPS Silver Staining

Bacteria were scraped off YESCA plates and resuspended in phosphate buffered saline (PBS) (pH 7.4) before normalization of optical density at 600 nm (OD_600_). To solubilize CsgA, samples were briefly treated with formic acid as described [2] or with hexafluoroisopronol (HFIP) [142]. Whole cell samples were electrophoresed on 13% sodium dodecyl sulfate (SDS)-polyacrylamide and blotted onto polyvinylidene difluoride using standard techniques. CsgA and CsgG polyclonal antibodies were raised in rabbits with the purified proteins (Proteintech, Chicago, IL, USA) and were used at 1:10,000 and 1:100,000 dilutions, respectively. The CsgD antibodies were kindly provided by Ute Romling and were used at a 1:5000 dilution. Goat anti-rabbit IgG-HRP (immunoglobulin G-horseradish peroxidase) (Sigma, St. Louis, MO, USA) was used at a 1:10,000 dilution for CsgA and CsgG blots and a 1:5,000 dilution for the CsgD blots. CsgD blots were transferred onto nitrocellulose membranes using pH 11.2 buffer containing 25 mM *N*-cyclohexyl-3-aminopropanesulfonic acid (CAPS) and 10% Methanol. Western blots were developed using the chemiluminiscent Pierce super signal detection system (Thermofisher Scientific, Waltham, MA, USA). LPS was extracted as described [143] from OD_600_ of 10 cells grown on YESCA plates for 48 h at 26 °C. Samples were normalized to 10 ng of keto-deoxy-d-manno-8-octanoic acid (KDO) per lane using the Thiobarbituric Acid Assay and were separated and silver stained in a 14% Tricine SDS-PAGE gel [143].

### 3.3. RNA Extraction

RNA extraction was performed as described [144] with modifications. Briefly, overnight cultures grown in LB media were normalized to an OD_600_ of 1 and 4 µL were plated on YESCA agar. Plates were incubated at 26 °C for 24 h. Colonies were harvested for RNA extraction by resuspension in a 2:1 solution of RNA protect (Qiagen, Hilden, Germany):YESCA. Cells were pelleted via centrifugation: 7000× *g* for 5 min at room temperature. Cell pellets were immediately frozen and stored at −80 °C. Pellets were thawed at room temperature and resuspended in RNA extraction solution (18 mM ethylenediaminetetraacetic acid (EDTA), 0.025% SDS, 1% β-mercaptoethanol, 95% formamide). Cells were lysed by incubation at 100 °C for 7 min. After centrifugation: 16,000× *g* for 5 min at room temperature, the supernatant was transferred to a new microcentrifuge tube. The solution was diluted with 4 volumes RNase-free H_2_O, and then 0.1 volume 3 M sodium acetate pH 5.2 and 2 volumes ethanol. The solution was placed at −80 °C for at least 1 h or overnight. Precipitated nucleic acids were pelleted via centrifugation: 16,000× *g* for 20 min at 4 °C and washed with ice-cold 75% ethanol. The pellets were resuspended in 1× DNase I buffer (New England Biolabs, Ipswich, Massachusetts, USA) and incubated at 55 °C for 10 min. The solutions were clarified via centrifugation: 16,000× *g* for 5 min at room temperature and the supernatant was transferred to a new microcentrifuge tube and digested with DNase I (NEB) for 45 min at 37 °C. RNA was precipitated by adding 0.1 volume 8 M LiCl and 3 volumes ethanol and placing at −80 °C overnight. RNA was pelleted via centrifugation: 16,000× *g* for 15 min at 4 °C and washed with ice-cold 75% ethanol. The RNA pellet was dried and resuspended in RNase free H_2_O.

### 3.4. Reverse Transcriptase Complementary DNA Synthesis and Real-Time Quantitative PCR

Reverse transcriptase complementary DNA (cDNA) synthesis was performed using Promega Go-Script (A5003) (Promega, Madison, WI, USA) according to the manufacturer’s instructions with random primers (Invitrogen, Carlsbad, CA, USA). Real-time quantitative PCR was performed using Invitrogen Power Sybr green master mix (Invitrogen, Carlsbad, CA, USA) according to the manufacturer’s instructions. Briefly, reverse transcriptase reactions were diluted 1000-fold for reactions using csgD primers and 10,000-fold for reactions using 16s primers. The data were analyzed using the Pfaffl method [145]. The amplification efficiencies of each primer pair were calculated via a standard dilution and relative RNA levels were calculated for each primer for each sample. Transcript levels of *csgD* were normalized to 16s levels.

### 3.5. Quantitative Real Time-PCR Analysis

The mRNA extraction was based on protocols for the RNeasy Mini Kit (Qiagen) with minor changes. Briefly, bacteria were normalized to an OD_600_ of 1 and 30 uL were spread onto YESCA plates and allowed to grow at 26 °C. After 24 h, bacteria were resuspended in 1.5 mL of RNAprotect (Qiagen) vortexed and incubated for 5 min. Cells were pelleted at 5000× *g* for 10 min and treated with lysozyme (Thermo Fisher Scientific, Waltham, MA, USA) (200 μL of freshly prepared 1mg/mL stock in 30 mM Tris-HCl 10 mM EDTA pH 8.0) for 5 min at room temperature. RNA was purified using the RNeasy Mini kit (Qiagen, Venlo, The Netherlands) and included an in column treatment with RNase free DNase (Qiagen, Venlo, The Netherlands). The RNA yield and purity were quantified in an Infinite 200 Pro NanoQuant Tecan reader (Tecan, Männedorf, Switzerland) and only samples with a 260/280 nm ratio equals or over 2 were further used. Aliquots of 2 ug/mL of mRNA were used to synthesize the cDNA using the Promega ImProm II reverse transcription kit and adding betaine 5 mM solution to the pre-annealing step with the random primers. Three serial dilutions (10^−1^, 10^−2^ and 10^−3^) by duplicate of each sample were analyzed using the ABsolute Blue QPCR SYBR Green Low ROX supermix from Thermo Scientific (Waltham, MA, USA) (3:5), the absolute quantification plate type and a standard 7500 run mode in an Applied Biosystems 7500 Fast Real-Time PCR System (Applied Biosystems, Foster City, CA, USA). The primers were designed using Primer 3 software, sequences are indicated in the Table S9, and were commercially acquired from Integrated DNA Technologies. Primers were used at 1.225 pM final concentrations each in 20 µL total reaction volume. As internal controls 16s and *rpoA* gene expression were measured, both worked similarly but 16s was more consistent and abundant, so 16s was used in the experiments described in this work. After confirming that the 16s primers and the target gene primers had similar efficiencies using the cycle threshold values generated from the ABI 7500 Fast System software analysis (Applied Biosystems, Foster City, CA, USA), all the calculations for the fold gene expression data were done applying the ∆∆Ct standard calculation.

### 3.6. Motility Assay

Cells were grown overnight in YESCA with appropriate antibiotics. Saturated cultures were diluted 1/100 in fresh YESCA and grown to mid log phase (OD_600_ of 0.3–0.6). Strains were normalized to 0.2 OD_600_ in YESCA and 2 uL were inoculated into 0.2% Agar YESCA motility plates. Plates were grown for 8 h at 26 °C. The strains tested for motility were also tested for growth rates in YESCA at 26 °C using a Klett meter (Kats Enterprises, Denton, TX, USA). No appreciable growth rate differences were measured.

### 3.7. Electron Microscopy

Bacteria were grown on YESCA plates for 48 h at 26 °C. Samples were resuspended in PBS and stained with 2% uranyl acetate as previously described [1]. Grids were viewed using a Phillips CM10 microscope (Philips Electron Optics, Eindhoven, The Netherlands).

## 4. Conclusions

More than 300 gene deletions that altered curli amyloid levels were identified in our CR screen. Several of the mutants focused on in this study are part of regulatory cascades, including stress response systems, which affect *csg* transcription. General themes arose for two major stress response systems, σ^S^ and Cpx. Mutations that that result in elevated σ^S^ levels produced more curli, while, those that induced σ^E^ or the Cpx system, produced less curli. An intact cell envelope was required to support curli biogenesis, as mutations in LPS and OPG resulted in less extracellular amyloid. Curli amyloid production was tied to key metabolic pathways such as the TCA cycle, the nucleotide synthesis pathways, and the catabolite utilization pathways. Collectively, the number and diversity of mutations that result in altered CR binding demonstrate that amyloid biogenesis is a complex and highly regulated developmental pathway in *E. coli*.

## Figures and Tables

**Table 1:** Primary cellular role of genes that affect curli production.

Function	COG Group	Number (% Total)	Genes (Dark Mutants Are Bold)
**Information Storage**			
Translation, ribosome structure, and biogenesis	J	13 (4.2)	*efp*, *miaA*, *pcnB*, *poxA*, *rbfA*, *rimK*, *rimM*, *rplA*, *rpsF*, *rpsT*, *rsgA*, *srmB*, *truB*
Transcription	K	43 (13.8)	*aaeR*, *arcA*, *asnC*, *cpxR*, *crp*, *cra*, *csgD*, *cusR*, *cysB*, *cytR*, *dksA*, *fliT*, *fhlA*, *flgM*, *gcvA*, *greA*, *hdfR*, *hfq*, *ihfA*, *ihfB*, *mlrA*, *mtlR*, *nagK*, *nanK*, *nusB*, *ompR*, *perR*, *purR*, *puuR*, *rcsB*, *rffC*, *rpoN*, *rpoS*, *rpoZ*, *rstA*, *sdiA*, *srlR*, *treR*, *waaH*, *xapR*, *ydcI*, *yieP*, *ynaK*
DNA replication, recombination, and repair	L	11 (3.5)	*atl*, *dam*, *dnaG*, *dnaT*, *ihfA*, *ihfB*, *nudC*, *nudL*, *priA*, *rnhA*, *rppH*
**Cellular processes**			
Cell envelope biogenesis, outer membrane	M	29 (9.3)	*csgA*, *csgB*, *csgE*, *csgF*, *csgG*, *cusB*, *galU*, *lpp*, *mdoH*, *mltA*, *mltB* , *nlpD*, *ompC*, *ompF*, *rcsF*, *pal*, *rfe*, *rffA*, *rffT*, *slp*, *tolc*, *waaC*, *waaD*, *waaE*, *waaF*, *waaG*, *waaP*, *wzxE*, *ycgV*
Cell motility and secretion	N	5 (1.6)	*cpxP*, *flgM*, *fliI*, *tolA*, *ycbR*
Posttranslational modification, protein turnover, chaperones	O	11 (3.5)	*ccmA*, *clpA*, *clpP*, *clpX*, *dnaK*, *lon*, *sspA*, *surA*, *yfgC*, *yjjW*, *yncG*
Inorganic ion transport and metabolism	P	14 (4.5)	*cpxP*, *cysC*, *cysI*, *ddpD*, *dps*, *fepB*, *fepC*, *fepD*, *fepG*, *fes*, *mdfA*, *mdoG*, *nhaA*, *yoeE*
Signal transduction mechanisms	T	23 (7.4)	*arcA*, *clpX*, *cpxA*, *cpxP*, *cpxR*, *crp*, *cusR*, *dksA*, *envZ*, *fhlA*, *gmr*, *kdpD*, *narQ*, *ompR*, *qseC*, *rseA*, *rstA*, *rstB*, *uspE*, *ydaM*, *yedV*, *yeiL*, *zraR*
Defense Mechanism	V	2 (0.6)	*cusB*, *yfeW*
**Metabolism**			
Energy production and conversion	C	37 (11.9)	*aceE*, *aegA*, *appC*, *aspA*, *atpA*, *atpB*, *atpC*, *atpE*, *atpF*, *atpG*, *atpH*, *dlgD*, *fadH*, *hybD*, *lpd*, *nuoA*, *nuoB*, *nuoC*, *nuoE*, *nuoF*, *nuoG*, *nuoJ*, *nuoK*, *nuoM*, *nuoN*, *putA*, *racC*, *sdhA*, *sdhB*, *sdhC*, *sdhD*, *sucA*, *sucC*, *ubiF*, *ubiH*, *ydiT*, *yjjW*
Amino acid transport and metabolism	E	28 (9.0)	*argC*, *aroA*, *aroB*, *aroC*, *aroD*, *aroE*, *aroH*, *aroK*, *aroP*, *aspA*, *aspC*, *carA*, *carB*, *cysE*, *ddpD*, *eamA*, *glnA*, *gmhB*, *mdfA*, *metC*, *pepT*, *proW*, *speB*, *trpA*, *trpB*, *trpC*, *trpD*, *trpE*
Nucleotide transport and metabolism	F	22 (7.1)	*carA*, *carB*, *cmk*, *cyaA*, *guaB*, *ndk*, *purA*, *purC*, *purD*, *purE*, *purF*, *purH*, *purK*, *purL*, *purM*, *purU*, *pyrB*, *pyrC*, *pyrD*, *pyrE*, *pyrF*, *thyA*
Carbohydrate transport and metabolism	G	21 (6.7)	*eamA*, *fbp*, *glmM*, *glvG*, *glxK*, *gmhA*, *gnd*, *lapB*, *lsrF*, *mdfA*, *nagA*, *nagC*, *nagK*, *nanE*, *nanK*, *pgm*, *ptsH*, *ptsI*, *rafD*, *tktA*, *tpiA*
Coenzyme metabolism	H	8 (3.8)	*cysG*, *fepC*, *lipA*, *lipB*, *pdxH*, *rimK*, *trpA*, *trpB*, *ubiE*, *ubiF*, *ubiG*, *ubiH*
Lipid metabolism	I	3 (1.0)	*fabF*, *fabH*, *fadJ*
Secondary metabolites	Q	2 (0.6)	*fabF*, *paaI*
**Poorly characterized**			
General function prediction	R	14 (4.5)	*essQ*, *ilvG*, *iptB*, *nudL*, *php*, *rapZ*, *rppH*, *uidC*, *ybcI*, *ybgC*, *ydeJ*, *yeiR*, *ygfZ*, *yheV*, *ynjD*, *yqaB*
Unknown/Other	S,U	22 (7.1)	*mdoC*, *rodZ*, *tolB*, *tolQ*, *tolR*, *tomb*, *torI*, *tpr*, *ybaM*, *ybaP*, *yciC*, *ydaF*, *ydaT*, *ydbH*, *yecH*, *yedQ*, *yeeN*, *yfcL*, *ygiU*, *yicC*, *ykgH*, *ymgE*

COG: clusters of orthologous groups.

**Table 2:** Selected mutant *csgD* levels as compared to WT.

CR Phenotype	Strain	Average Relative to BW25113 (WT)	Standard Deviation	Outcome of *t*-Test
Red	BW25113	1	N/A	N/A
White	*csgD*	N.D.	N/A	N/A
White	*nhaA*	0.018	±0.0068	*p*-value < 0.001
White	*php*	0.841	±0.130	Not significant
White	*purD*	0.036	±0.053	*p*-value < 0.001
White	*lon*	0.392	±0.187	*p-*value < 0.05
Light Pink	*waaC*	1.118	±0.363	Not significant
Light Pink	*dnaK*	0.221	±0.034	*p*-value < 0.001
Light Pink	*speB*	0.578	±0.114	*p*-value < 0.05
Light Pink	*hfq*	0.06	±0.080	*p*-value < 0.001
Light Pink	*aroA*	0.223	±0.057	*p*-value < 0.05
Pink	*fabH*	1.171	±0.315	Not significant
Pink	*flgM*	0.86	±0.254	Not significant
Pink	*ddpD*	0.766	±0.337	Not significant
Pink	*pyrC*	0.045	±0.020	*p*-value < 0.001
Pink	nagA *	3.38	±1.946	*p*-value < 0.05
Pink	*fhlA*	0.695	±0.132	*p*-value < 0.05
Pink	dksA *	5.115	±1.076	*p*-value < 0.001
Pink	*hybD*	0.278	±0.043	*p*-value < 0.05
Pink	*rstA*	0.492	±0.187	*p*-value < 0.05
Light Red	*priA*	0.023	±0.004	*p*-value < 0.001
Light Red	*aaeR*	2.324	±0.755	*p*-value < 0.05
Light Red	*glvG*	1.316	±0.175	Not significant
Light Red	*cmr*	1.586	±0.561	Not significant
Light Red	*dam*	0.967	±0.268	Not significant
Light Red	*hdfR*	0.752	±0.254	Not significant
Light Red	*mltA*	0.874	±0.170	Not significant
Dark Red	*cysB*	3.06	±0.982	*p*-value < 0.001
Dark Red	*pcnB*	1.602	±0.354	*p*-value < 0.05
Dark Red	*truB*	1.371	±0.240	Not significant
Dark Red	*rcsB*	3.723	±0.970	*p*-value < 0.05
Dark Red	*sdiA*	2.952	±0.684	*p*-value < 0.05
Dark Red	*fes*	0.999	±0.217	Not significant
Dark Red	*nuoA*	2.302	±0.678	Not significant
Dark Red	*qseC*	2.861	±2.190	Not significant
Dark Red	*arcA*	1.603	±0.219	*p*-value < 0.05
Dark Red	*mdoC*	1.581	±0.309	*p*-value < 0.05
Dark Red	*perR*	1.923	±0.537	*p*-value < 0.05
Dark Red	*cusB*	2.224	±0.998	*p*-value < 0.05

* Increased *csgD* transcript levels driven by decrease in 16s ribosomal RNA (rRNA) levels (see Table 4). CR denotes Congo red (CR) binding phenotype.

**Table 3:** *csgD* qualitative PCR significance for selected mutants.

Outcome of *t*-Test	Number of Strains
Not significant	15
*p*-value < 0.05	14
*p*-value < 0.001	7
Uncertain	2
Total	38

**Table 4:** *csgD* levels altered by decreased ribosome RNA levels.

Strain	Aveage *csgD* Levels	Std Dev	*p*-Value	Average *16s* Levels	Std Dev	*p-*Value
BW25113	3.141 × 10^−2^	1.652 × 10^−2^	N/A	1.38 × 10^−2^	7.56 × 10^−3^	N/A
*dam*	4.605 × 10^−2^	1.173 × 10^−2^	0.107	2.05 × 10^−2^	4.31 × 10^−3^	8.93 × 10^−2^
*hfq*	1.742 × 10^−4^	3.279 × 10^−5^	9.36 × 10^−4^	6.46 × 10^−3^	6.48 × 10^−3^	1.02 × 10^−1^
*nagA*	2.407 × 10^−2^	2.143 × 10^−3^	0.306	4.79 × 10^−3^	4.23 × 10^−3^	2.94 × 10^−2^
*dksA*	3.777 × 10^−2^	9.779 × 10^−3^	0.436	3.14 × 10^−3^	8.50 × 10^−4^	6.51× 10^−3^

Std Dev: Standard deviation.

## Supplementary Materials

The following are available online at www.mdpi.com/2218-273X/7/4/75/s1, Figure S1: Congo Red and hydrophobicity of different LPS mutant *E. coli* strains, Figure S2: Differences in Congo Red phenotypes of Keio collection strains on YESCA and CFA plates and CFA plates without Coommassie Brilliant Blue counterstain, Figure S3: qRT-PCR of inner core LPS and *nhaA* mutants, Figure S4: Effects of σE induction on curli production, Figure S5: The intergenic region between *csgD* and *csgB* has many transcriptional binding sites, Figure S6: Low ppGpp strains and *dksA* mutants produce less curli, Table S1: Congo red phenotypes and Western blot data from Keio strains with altered curli production, Table S2: Phenotypes of suppressor strains with more than one CR phenotype and the primers used, Table S3: Cellular localization of identified gene products, Table S4: Genes required for or known to affect curli production, Table S5: CsgD regulon excluding c*sgBA*C and cs*gDEF*G, Table S6: Intergenic regions in *E. coli* and the number of transcriptional binding sites in divergent intergenic regions, Table S7: Venn diagram gene List, Table S8: Congo red phenotypes of Keio strains on YESCA and CFA plates or CFA plates without Coommassie Brilliant Blue, Table S9: CsgD protein and transcript levels relative to WT, Table S10: Strains, plasmids, and primers used.
