# Supplementary material for: The Production of Curli Amyloid Fibers Is Deeply Integrated into the Biology of Escherichia coli
Source: Biomolecules. 2017 Oct 31;7(4):75. doi: 10.3390/biom7040075 (PMC5745457; doi:10.3390/biom7040075)
Supplement: Supplementary file 1 [file biomolecules-07-00075-s001.zip › biomolecules-227744-SuppFigures resubmit.docx]

**Supplementary Materials**

**Fig. S1. Congo Red and hydrophobicity of different LPS mutant *E. coli* strains. A**. Congo Red indicator plates of WT (BW25113), *csgA*, *galU*, *waaC*, *waaF*, and *waaG* grown for 2 days at 26°C. A sterile loop was scraped through each strain to show the dry, friable phenotypes. **B**. Cell hydrophobicity of LPS mutant strains in phosphate buffered saline (PBS) amended with 2 M ammonium sulfate. Similar results were obtained in independent replicates (data not shown).

**Fig. S2. Differences in Congo Red phenotypes of Keio collection strains on YESCA and CFA plates and CFA plates without Coommassie Brilliant Blue counterstain.** Niba et al 2007 (46) performed a screen of the Keio collection for biofilm defective mutants. The biofilm mutants were subsequently tested for their motility, Type I pili formation, and curli production. For curli production they grew strains on CFA agar with twice the usual amount of Congo Red and no Coommassie Brilliant Blue (CBB) counterstain. Some of their CR phenotypes which were scored as +, -, or ± were different than ours. We tested these strains on YESCA and CFA plates with CR and CBB and on CFA plates without CBB and twice the CR as in Niba et al 2007 (46). Sets (A) (B) and (C) were scored based on their CR phenotypes from 1-6 at 24, 48, and 72 hours at 26⁰C (See Table S8). Shown here is 48 hours growth. Some of the phenotypic differences are due to plating differences. CBB aids in detecting subtle differences between strains. The *crp* shown in (C) was found to have a suppressor; a new mutant had a white CR phenotype like *cyaA.*

**Fig. S3. qRT-PCR of inner core LPS and nhaA mutants.** Relative levels of the indicated transcripts to BW25113 (WT) following 24 hours growth at 26°C as measured by RT-PCR. *csgA* and *csgD* transcript levels were graphed. Error bars are standard error of the mean of at least five measurements. * indicates p < 0.05 from student t-test*.*


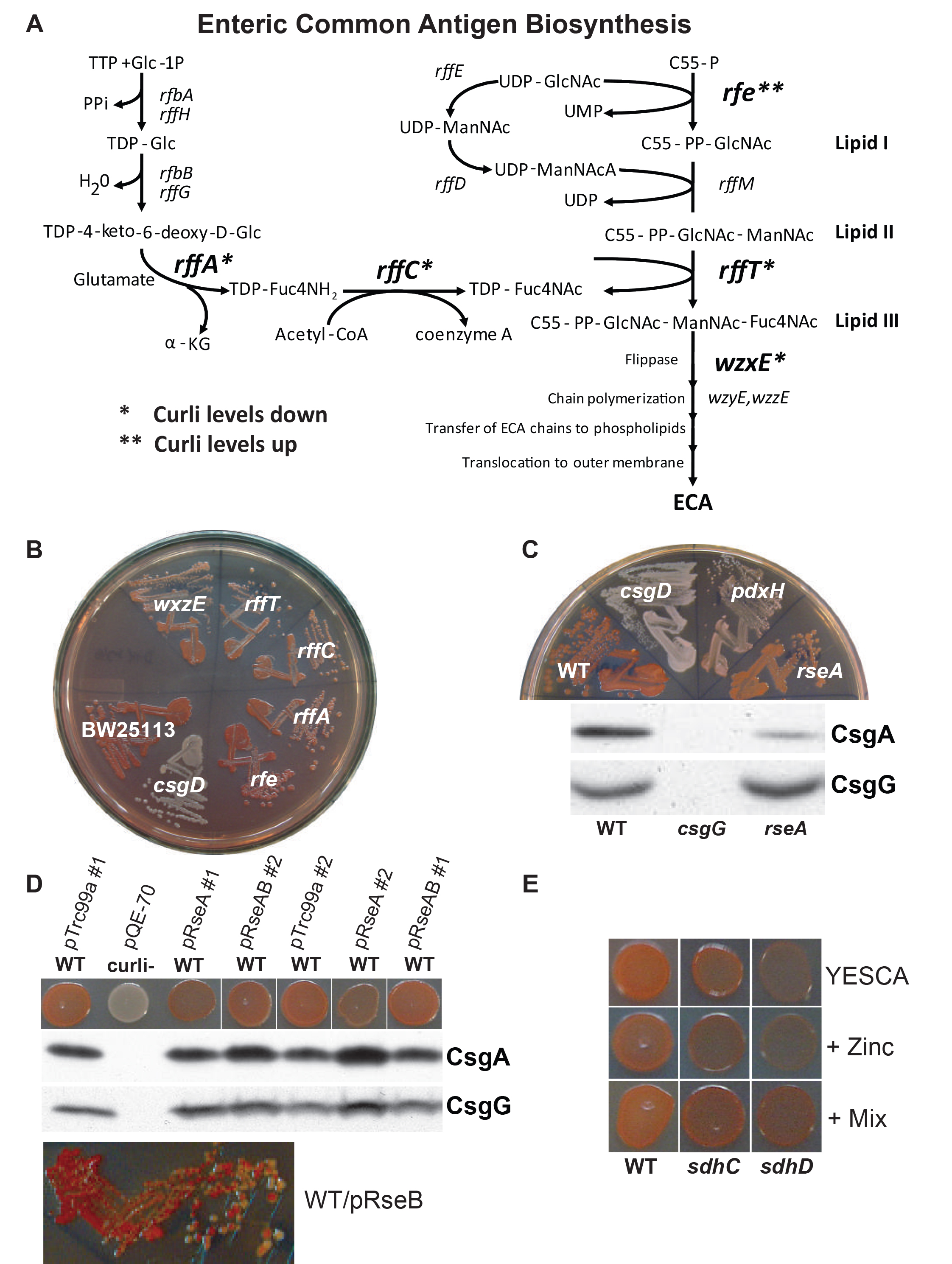


**Fig. S4. Effects of σ^E^ induction on curli production.** **A**. Diagram of Enteric Common Antigen biosynthetic pathway. **B**. Curli deficient strains: *rffA, rffC, rffT, wxzE*. Strains with increased curli production: *rfe.* **C**. Congo Red binding and whole cell Western blots of BW25113, *csgG*, and *rseA* mutants probed with antibodies to CsgA and CsgG. The *rseA* strain has a mucoid phenotype. **D**. Expression of *rseA in trans* using pRseA or pRseAB initially increased curli production in BW25113; however, these strains often reverted to normal curli production or variable CR phenotypes and colony morphologies (data not shown). When WT was transformed with pRseB, both pink and white colonies appeared in a near equal ratio of pink to red. When either colony phenotype was streaked again, both phenotypes appeared with a slight bias towards the original color. The streak shown was originally a red colony that arose from streaking a pink colony. Retransformation of pRseB plasmids obtained from different colony phenotypes displayed similar mixed phenotypes. **E**. Addition of ZnCl2 (0.25mM) or divalent mix to CR indicator plates partially rescues the curli defect in *sdhA* and *sdhB.*


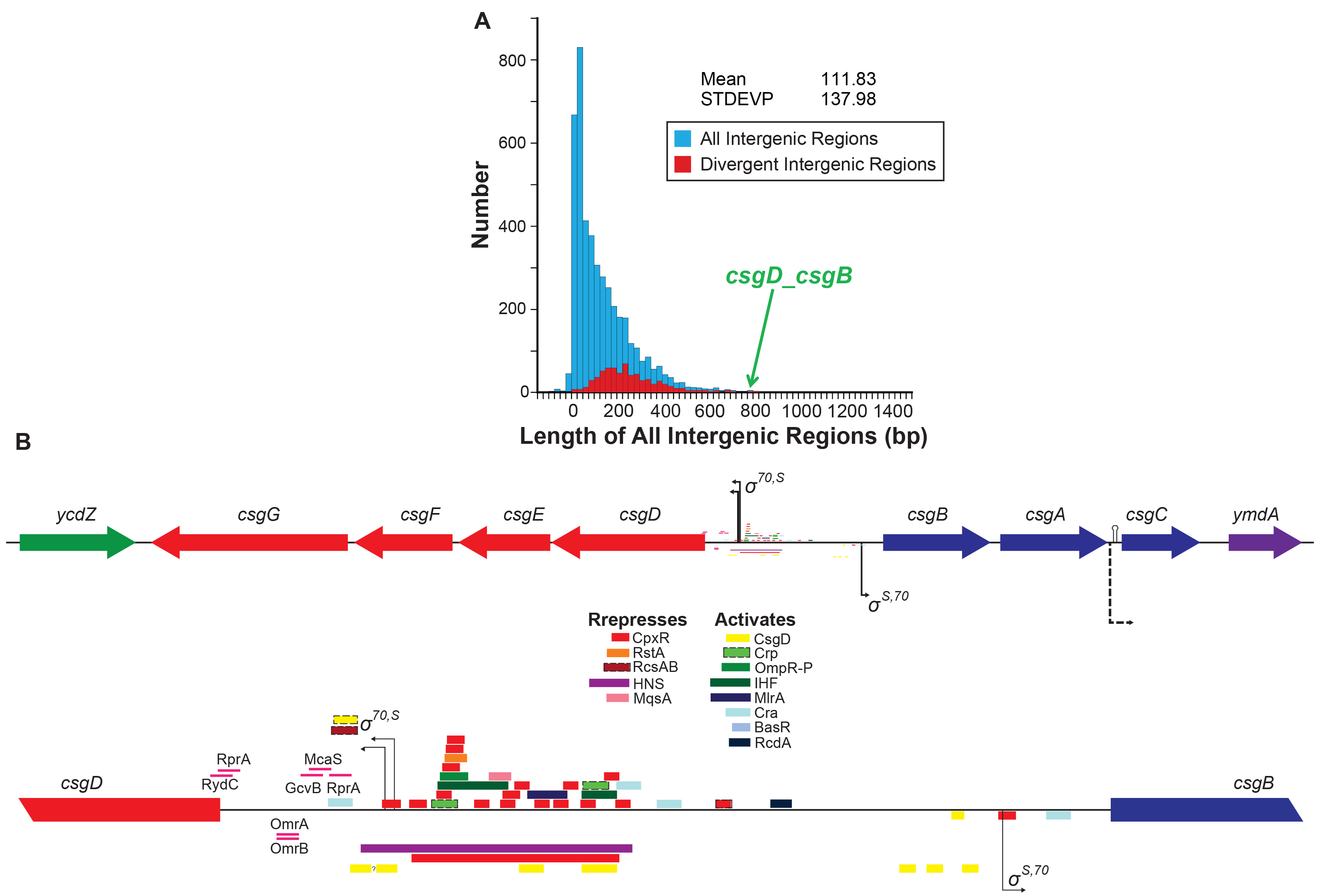


**Fig. S5. The intergenic region between *csgD* and *csgB* has many transcriptional binding sites. A**. Histogram of the length of all intergenic regions (blue) with an overlay of histogram of divergent intergenic regions (red). Divergent intergenic regions are shifted to larger sizes. **B**. Genomic context of *csg* genes (top) and the mapped transcriptional binding sites for the intergenic region between *csgD* and *csgB* (bottom) to scale. The scale of the intergenic region is one base pair per 0.25mm. Binding sites with number designations are from Ecocyc; the labeled CsgD and RcsAB binding sites near the transcription start of the *csgDEFG* operon have not been mapped (48). CpxR sites are depicted as separate binding sites at Ecocyc; however, they were originally a single large CpxR binding region. This large CpxR binding site and large HNS binding site are not currently annotated in Ecocyc (48, 49). Dan is also proposed to bind in the intergenic region and in the *csgB* ORF; however, the exact site or sites have not been mapped (65). The HNS and IHF binding sites may be multiple individual sites; their large size is much greater than their consensus sequences of 10 and 13bp, respectively (39).


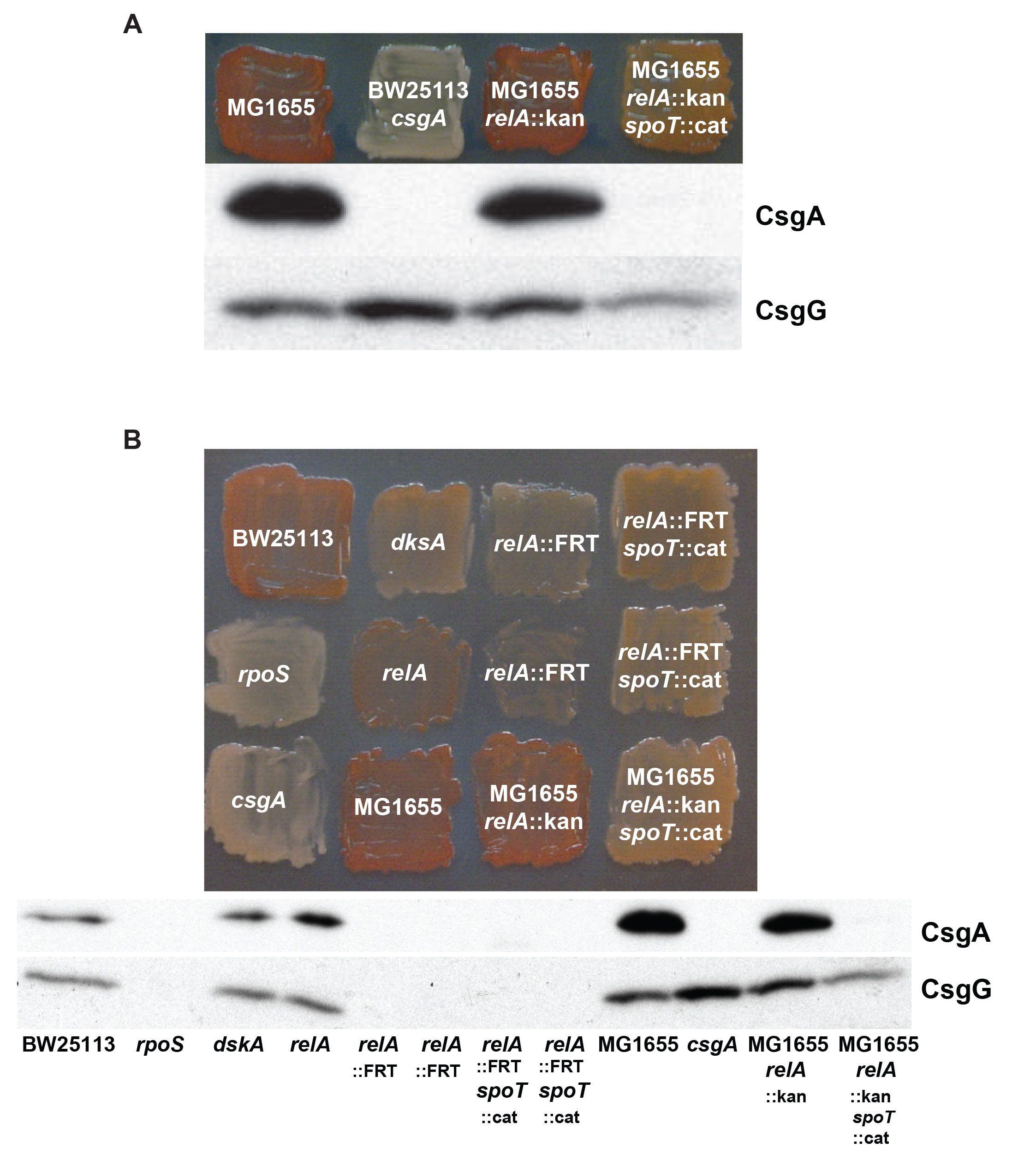


**Fig. S6. Low ppGpp strains and *dksA* mutants produce less curli.** Congo Red binding and Western blots of strains deficient for producing ppGpp. **A**. Background is *E. coli* strain BW25113 (6) unless noted as MG1655. Strain *relA*::FRT has had the kanamycin cassette of the *relA* Keio strain (*relA*::FRT-*kan-*FRT) excised using pCP20 which encodes Flp recombinase (21). **B**. The FRT clean deletions were compared to keio collection strains. The *spoT*::cat (*∆spoT207*::cat) was moved into the *relA*::FRT strain by P1 transduction; however, the *relA*::FRT strain is more defective than the *relA* Keio strain. The duplicate strains of *relA*::FRT and *relA::FRT spot*::cat shown were independent isolates. MG1655 *relA*::kan denotes MG1655 *relA*251::kan. Strains were grown on YESCA plates for two days at 26°C.

Supplemental methods

PCR verification of strains with multiple CR phenotypes.

Strains with multiple phenotypes were chosen for PCR verification. Primers were designed 2-300 bp upstream of each gene and are listed in Table S4. Each strain with an altered CR phenotype was struck from the Keio collection to make a clean freezer stock. At least two independent colonies for each phenotype were resuspended in sterile water and subjected to colony PCR. The individual colony mixtures were added to master mixes of GoTaq Flexi (Promega). Mixture A contained the appropriate upstream primer and primer K1 (6) and was used to verify the location of the kanamycin insert in the genome. Mixture B contained the primers KT and K2 (6) and was used to verify the presence of the insert. If each phenotype gave a positive PCR product for both mixtures then it was added to Table S2. Mutations in energy production, coenzyme metabolism, cell envelope biogenesis, and DNA repair genes were more likely to have more than one CR phenotype (Table S2).

Cell hydrophobicity.

Cell hydrophobicity was measured as described (59) using Xylene (X5-1; Fisher Scientific Co., Pittsburgh, PA), which gave more consistent results than a mixture of linear hexanes (74). The assay was performed with 2 mL of cells resuspended to 1 OD600 in PBS pH 7.4 amended with 2 M ammonium sulfate (57, 58), which helps distinguish between the relatively hydrophilic K-12 strains. Cells were tested in 16x125 mm glass culture tubes overlaid with increasing amounts of Xylene. Following incubation for 10 minutes at room temperature, the mixtures were vortexed for 1 minute using a Barnstead Thermolyne 16700. Following phase separation for 15 minutes at room temperature, a sample from the lower aqueous layer was removed and measured at OD600. Each strain was independently tested at least twice.

References

1. **Ahuja, N., D. Korkin, R. Chaba, B. O. Cezairliyan, R. T. Sauer, K. K. Kim, and C. A. Gross.** 2009. Analyzing the interaction of RseA and RseB, the two negative regulators of the sigmaE envelope stress response, using a combined bioinformatic and experimental strategy. J Biol Chem **284:**5403-13.

2. **Amann, E., B. Ochs, and K. J. Abel.** 1988. Tightly regulated tac promoter vectors useful for the expression of unfused and fused proteins in *Escherichia coli*. Gene **69:**301-15.

3. **Anriany, Y., S. N. Sahu, K. R. Wessels, L. M. McCann, and S. W. Joseph.** 2006. Alteration of the rugose phenotype in waaG and ddhC mutants of *Salmonella enterica serovar Typhimurium* DT104 is associated with inverse production of curli and cellulose. Appl Environ Microbiol **72:**5002-12.

4. **Arnqvist, A., A. Olsen, and S. Normark.** 1994. Sigma S-dependent growth-phase induction of the csgBA promoter in *Escherichia coli* can be achieved in vivo by sigma 70 in the absence of the nucleoid-associated protein H-NS. Mol Microbiol **13:**1021-32.

5. **Arnqvist, A., A. Olsen, J. Pfeifer, D. G. Russell, and S. Normark.** 1992. The Crl protein activates cryptic genes for curli formation and fibronectin binding in *Escherichia coli* HB101. Mol Microbiol **6:**2443-52.

6. **Baba, T., T. Ara, M. Hasegawa, Y. Takai, Y. Okumura, M. Baba, K. A. Datsenko, M. Tomita, B. L. Wanner, and H. Mori.** 2006. Construction of *Escherichia coli* K-12 in-frame, single-gene knockout mutants: the Keio collection. Mol Syst Biol **2:**2006 0008.

7. **Barnhart, M. M., J. Lynem, and M. R. Chapman.** 2006. GlcNAc-6P levels modulate the expression of Curli fibers by *Escherichia coli*. J Bacteriol **188:**5212-9.

8. **Blattner, F. R., G. Plunkett, 3rd, C. A. Bloch, N. T. Perna, V. Burland, M. Riley, J. Collado-Vides, J. D. Glasner, C. K. Rode, G. F. Mayhew, J. Gregor, N. W. Davis, H. A. Kirkpatrick, M. A. Goeden, D. J. Rose, B. Mau, and Y. Shao.** 1997. The complete genome sequence of *Escherichia coli* K-12. Science **277:**1453-62.

9. **Bougdour, A., C. Lelong, and J. Geiselmann.** 2004. Crl, a low temperature-induced protein in *Escherichia coli* that binds directly to the stationary phase sigma subunit of RNA polymerase. J Biol Chem **279:**19540-50.

10. **Brombacher, E., A. Baratto, C. Dorel, and P. Landini.** 2006. Gene expression regulation by the Curli activator CsgD protein: modulation of cellulose biosynthesis and control of negative determinants for microbial adhesion. J Bacteriol **188:**2027-37.

11. **Brombacher, E., C. Dorel, A. J. Zehnder, and P. Landini.** 2003. The curli biosynthesis regulator CsgD co-ordinates the expression of both positive and negative determinants for biofilm formation in *Escherichia coli*. Microbiology **149:**2847-57.

12. **Brown, P. K., C. M. Dozois, C. A. Nickerson, A. Zuppardo, J. Terlonge, and R. Curtiss, 3rd.** 2001. MlrA, a novel regulator of curli (AgF) and extracellular matrix synthesis by Escherichia coli and Salmonella enterica serovar Typhimurium. Mol Microbiol **41:**349-63.

13. **Campbell, A.** 1961. Sensitive mutants of bacteriophage lambda. Virology **14:**22-32.

14. **Casadaban, M. J.** 1976. Transposition and fusion of the lac genes to selected promoters in Escherichia coli using bacteriophage lambda and Mu. J Mol Biol **104:**541-55.

15. **Chapman, M. R., L. S. Robinson, J. S. Pinkner, R. Roth, J. Heuser, M. Hammar, S. Normark, and S. J. Hultgren.** 2002. Role of *Escherichia coli* curli operons in directing amyloid fiber formation. Science **295:**851-5.

16. **Chen, S. L., C. S. Hung, J. Xu, C. S. Reigstad, V. Magrini, A. Sabo, D. Blasiar, T. Bieri, R. R. Meyer, P. Ozersky, J. R. Armstrong, R. S. Fulton, J. P. Latreille, J. Spieth, T. M. Hooton, E. R. Mardis, S. J. Hultgren, and J. I. Gordon.** 2006. Identification of genes subject to positive selection in uropathogenic strains of *Escherichia coli*: a comparative genomics approach. Proc Natl Acad Sci U S A **103:**5977-82.

17. **Chirwa, N. T., and M. B. Herrington.** 2003. CsgD, a regulator of curli and cellulose synthesis, also regulates serine hydroxymethyltransferase synthesis in *Escherichia coli* K-12. Microbiology **149:**525-35.

18. **Chirwa, N. T., and M. B. Herrington.** 2004. Role of MetR and PurR in the activation of *glyA* by CsgD in *Escherichia coli* K-12. Can J Microbiol **50:**683-90.

19. **Costanzo, A., and S. E. Ades.** 2006. Growth phase-dependent regulation of the extracytoplasmic stress factor, sigmaE, by guanosine 3',5'-bispyrophosphate (ppGpp). J Bacteriol **188:**4627-34.

20. **Da Re, S., and J. M. Ghigo.** 2006. A CsgD-independent pathway for cellulose production and biofilm formation in *Escherichia coli*. J Bacteriol **188:**3073-87.

21. **Datsenko, K. A., and B. L. Wanner.** 2000. One-step inactivation of chromosomal genes in *Escherichia coli* K-12 using PCR products. Proc Natl Acad Sci U S A **97:**6640-5.

22. **De Las Penas, A., L. Connolly, and C. A. Gross.** 1997. The sigmaE-mediated response to extracytoplasmic stress in *Escherichia coli* is transduced by RseA and RseB, two negative regulators of sigmaE. Mol Microbiol **24:**373-85.

23. **Dorel, C., O. Vidal, C. Prigent-Combaret, I. Vallet, and P. Lejeune.** 1999. Involvement of the Cpx signal transduction pathway of *E. coli* in biofilm formation. FEMS Microbiol Lett **178:**169-75.

24. **Epstein, E. A., M. A. Reizian, and M. R. Chapman.** 2009. Spatial clustering of the curlin secretion lipoprotein requires curli fiber assembly. J Bacteriol **191:**608-15.

25. **Ferrieres, L., and D. J. Clarke.** 2003. The RcsC sensor kinase is required for normal biofilm formation in *Escherichia coli* K-12 and controls the expression of a regulon in response to growth on a solid surface. Mol Microbiol **50:**1665-82.

26. **Gerstel, U., C. Park, and U. Romling.** 2003. Complex regulation of *csgD* promoter activity by global regulatory proteins. Mol Microbiol **49:**639-54.

27. **Gibson, D. L., A. P. White, C. M. Rajotte, and W. W. Kay.** 2007. AgfC and AgfE facilitate extracellular thin aggregative fimbriae synthesis in *Salmonella enteritidis*. Microbiology **153:**1131-40.

28. **Gibson, D. L., A. P. White, S. D. Snyder, S. Martin, C. Heiss, P. Azadi, M. Surette, and W. W. Kay.** 2006. *Salmonella* produces an O-antigen capsule regulated by AgfD and important for environmental persistence. J Bacteriol **188:**7722-30.

29. **Gualdi, L., L. Tagliabue, and P. Landini.** 2007. Biofilm formation-gene expression relay system in *Escherichia coli*: modulation of sigmaS-dependent gene expression by the CsgD regulatory protein via sigmaS protein stabilization. J Bacteriol **189:**8034-43.

30. **Hammar, M.** 1997 Assembly and adhesive properties of curli. Ph.D. thesis at Karolinska Institute, Stockholm, Sweden.

31. **Hammar, M., A. Arnqvist, Z. Bian, A. Olsen, and S. Normark.** 1995. Expression of two csg operons is required for production of fibronectin- and congo red-binding curli polymers in *Escherichia coli* K-12. Mol Microbiol **18:**661-70.

32. **Holmqvist, E., J. Reimegard, M. Sterk, N. Grantcharova, U. Romling, and E. G. Wagner.** 2010. Two antisense RNAs target the transcriptional regulator CsgD to inhibit curli synthesis. Embo J **29:**1840-50.

33. **Inoue, T., R. Shingaki, S. Hirose, K. Waki, H. Mori, and K. Fukui.** 2007. Genome-wide screening of genes required for swarming motility in *Escherichia coli* K-12. J Bacteriol **189:**950-7.

34. **Ishihama, A.** 2010. Prokaryotic genome regulation: multifactor promoters, multitarget regulators and hierarchic networks. FEMS Microbiol Rev.

35. **Jensen, K. F.** 1993. The *Escherichia coli* K-12 "wild types" W3110 and MG1655 have an *rph* frameshift mutation that leads to pyrimidine starvation due to low *pyrE* expression levels. J Bacteriol **175:**3401-7.

36. **Jiang, M., S. M. Sullivan, P. K. Wout, and J. R. Maddock.** 2007. G-protein control of the ribosome-associated stress response protein SpoT. J Bacteriol **189:**6140-7.

37. **Jubelin, G., A. Vianney, C. Beloin, J. M. Ghigo, J. C. Lazzaroni, P. Lejeune, and C. Dorel.** 2005. CpxR/OmpR interplay regulates curli gene expression in response to osmolarity in *Escherichia coli*. J Bacteriol **187:**2038-49.

38. **Kader, A., R. Simm, U. Gerstel, M. Morr, and U. Romling.** 2006. Hierarchical involvement of various GGDEF domain proteins in rdar morphotype development of S*almonella enterica serovar Typhimurium*. Mol Microbiol **60:**602-16.

39. **Karp, P. D., I. M. Keseler, A. Shearer, M. Latendresse, M. Krummenacker, S. M. Paley, I. Paulsen, J. Collado-Vides, S. Gama-Castro, M. Peralta-Gil, A. Santos-Zavaleta, M. I. Penaloza-Spinola, C. Bonavides-Martinez, and J. Ingraham.** 2007. Multidimensional annotation of the *Escherichia coli* K-12 genome. Nucleic Acids Res **35:**7577-90.

40. **Kim, S. H., and Y. H. Kim.** 2004. *Escherichia coli* O157:H7 adherence to HEp-2 cells is implicated with curli expression and outer membrane integrity. J Vet Sci **5:**119-24.

41. **Kostakioti, M., M. Hadjifrangiskou, J. S. Pinkner, and S. J. Hultgren.** 2009. QseC-mediated dephosphorylation of QseB is required for expression of genes associated with virulence in uropathogenic Escherichia coli. Mol Microbiol **73:**1020-31.

42. **Latasa, C., A. Roux, A. Toledo-Arana, J. M. Ghigo, C. Gamazo, J. R. Penades, and I. Lasa.** 2005. BapA, a large secreted protein required for biofilm formation and host colonization of *Salmonella enterica serovar Enteritidis*. Mol Microbiol **58:**1322-39.

43. **Lee, J., T. Maeda, S. H. Hong, and T. K. Wood.** 2009. Reconfiguring the quorum-sensing regulator SdiA of *Escherichia coli* to control biofilm formation via indole and N-acylhomoserine lactones. Appl Environ Microbiol **75:**1703-16.

44. **Mulvey, M. A., Y. S. Lopez-Boado, C. L. Wilson, R. Roth, W. C. Parks, J. Heuser, and S. J. Hultgren.** 1998. Induction and evasion of host defenses by type 1-piliated uropathogenic *Escherichia coli*. Science **282:**1494-7.

45. **Nenninger, A. A., L. S. Robinson, and S. J. Hultgren.** 2009. Localized and efficient curli nucleation requires the chaperone-like amyloid assembly protein CsgF. Proc Natl Acad Sci U S A **106:**900-5.

46. **Niba, E. T., Y. Naka, M. Nagase, H. Mori, and M. Kitakawa.** 2007. A genome-wide approach to identify the genes involved in biofilm formation in *E. coli*. DNA Res **14:**237-46.

47. **Ogasawara, H., A. Hasegawa, E. Kanda, T. Miki, K. Yamamoto, and A. Ishihama.** 2007. Genomic SELEX search for target promoters under the control of the PhoQP-RstBA signal relay cascade. J Bacteriol **189:**4791-9.

48. **Ogasawara, H., K. Yamada, A. Kori, K. Yamamoto, and A. Ishihama.** 2010. Regulation of the *E. coli* *csgD* Promoter: Interplay between Five Transcription Factors. Microbiology.

49. **Ogasawara, H., K. Yamamoto, and A. Ishihama.** 2011. Role of the Biofilm Master Regulator CsgD in Cross-Regulation between Biofilm Formation and Flagellar Synthesis. J Bacteriol **193:**2587-97.

50. **Olsen, A., A. Arnqvist, M. Hammar, S. Sukupolvi, and S. Normark.** 1993. The RpoS sigma factor relieves H-NS-mediated transcriptional repression of *csgA*, the subunit gene of fibronectin-binding curli in *Escherichia coli*. Mol Microbiol **7:**523-36.

51. **Pesavento, C., G. Becker, N. Sommerfeldt, A. Possling, N. Tschowri, A. Mehlis, and R. Hengge.** 2008. Inverse regulatory coordination of motility and curli-mediated adhesion in *Escherichia coli*. Genes Dev **22:**2434-46.

52. **Peters, J. E., T. E. Thate, and N. L. Craig.** 2003. Definition of the *Escherichia coli* MC4100 genome by use of a DNA array. J Bacteriol **185:**2017-21.

53. **Pratt, L. A., and T. J. Silhavy.** 1998. Crl stimulates RpoS activity during stationary phase. Mol Microbiol **29:**1225-36.

54. **Prigent-Combaret, C., E. Brombacher, O. Vidal, A. Ambert, P. Lejeune, P. Landini, and C. Dorel.** 2001. Complex regulatory network controls initial adhesion and biofilm formation in *Escherichia coli* via regulation of the *csgD* gene. J Bacteriol **183:**7213-23.

55. **Romling, U., Z. Bian, M. Hammar, W. D. Sierralta, and S. Normark.** 1998. Curli fibers are highly conserved between *Salmonella typhimurium* and *Escherichia coli* with respect to operon structure and regulation. J Bacteriol **180:**722-31.

56. **Romling, U., M. Rohde, A. Olsen, S. Normark, and J. Reinkoster.** 2000. AgfD, the checkpoint of multicellular and aggregative behaviour in *Salmonella typhimurium* regulates at least two independent pathways. Mol Microbiol **36:**10-23.

57. **Rosenberg, M.** 1984. Ammonium sulphate enhances adherence of *Escherichia coli* J-5 to hydrocarbon and polystyrene, p. 41-45. FEMS Microbiol. Lett., vol. 25.

58. **Rosenberg, M.** 2006. Microbial adhesion to hydrocarbons: twenty-five years of doing MATH. FEMS Microbiol Lett **262:**129-34.

59. **Rosenberg, M., D. Gutnick, and E. Rosenberg.** 1980. Adherence of bacteria to hydrocarbons: a simple method for measuring cell surface hydrophobicity. FEMS Microbiol. Lett. **9:**29-33.

60. **Saldana, Z., J. Xicohtencatl-Cortes, F. Avelino, A. D. Phillips, J. B. Kaper, J. L. Puente, and J. A. Giron.** 2009. Synergistic role of curli and cellulose in cell adherence and biofilm formation of attaching and effacing *Escherichia coli* and identification of Fis as a negative regulator of curli. Environ Microbiol **11:**992-1006.

61. **Simm, R., A. Lusch, A. Kader, M. Andersson, and U. Romling.** 2007. Role of EAL-containing proteins in multicellular behavior of *Salmonella enterica serovar Typhimurium*. J Bacteriol **189:**3613-23.

62. **Solano, C., B. Garcia, C. Latasa, A. Toledo-Arana, V. Zorraquino, J. Valle, J. Casals, E. Pedroso, and I. Lasa.** 2009. Genetic reductionist approach for dissecting individual roles of GGDEF proteins within the c-di-GMP signaling network in *Salmonella*. Proc Natl Acad Sci U S A **106:**7997-8002.

63. **Sommerfeldt, N., A. Possling, G. Becker, C. Pesavento, N. Tschowri, and R. Hengge.** 2009. Gene expression patterns and differential input into curli fimbriae regulation of all GGDEF/EAL domain proteins in *Escherichia coli*. Microbiology **155:**1318-31.

64. **Stenutz, R., A. Weintraub, and G. Widmalm.** 2006. The structures of *Escherichia coli* O-polysaccharide antigens. FEMS Microbiol Rev **30:**382-403.

65. **Teramoto, J., S. H. Yoshimura, K. Takeyasu, and A. Ishihama.** 2010. A novel nucleoid protein of *Escherichia coli* induced under anaerobiotic growth conditions. Nucleic Acids Res **38:**3605-18.

66. **Tschowri, N., S. Busse, and R. Hengge.** 2009. The BLUF-EAL protein YcgF acts as a direct anti-repressor in a blue-light response of *Escherichia coli*. Genes Dev **23:**522-34.

67. **Uhlich, G. A., N. W. t. Gunther, D. O. Bayles, and D. A. Mosier.** 2009. The CsgA and Lpp proteins of an *Escherichia coli* O157:H7 strain affect HEp-2 cell invasion, motility, and biofilm formation. Infect Immun **77:**1543-52.

68. **Vianney, A., G. Jubelin, S. Renault, C. Dorel, P. Lejeune, and J. C. Lazzaroni.** 2005. *Escherichia coli* *tol* and *rcs* genes participate in the complex network affecting curli synthesis. Microbiology **151:**2487-97.

69. **Vidal, O., R. Longin, C. Prigent-Combaret, C. Dorel, M. Hooreman, and P. Lejeune.** 1998. Isolation of an *Escherichia coli* K-12 mutant strain able to form biofilms on inert surfaces: involvement of a new *ompR* allele that increases curli expression. J Bacteriol **180:**2442-9.

70. **Weber, H., C. Pesavento, A. Possling, G. Tischendorf, and R. Hengge.** 2006. Cyclic-di-GMP-mediated signalling within the sigma network of *Escherichia coli*. Mol Microbiol **62:**1014-34.

71. **White, A. P., A. M. Weljie, D. Apel, P. Zhang, R. Shaykhutdinov, H. J. Vogel, and M. G. Surette.** 2010. A global metabolic shift is linked to S*almonell*a multicellular development. PLoS ONE **5:**e11814.

72. **Xiao, H., M. Kalman, K. Ikehara, S. Zemel, G. Glaser, and M. Cashel.** 1991. Residual guanosine 3',5'-bispyrophosphate synthetic activity of *relA* null mutants can be eliminated by *spoT* null mutations. J Biol Chem **266:**5980-90.

73. **Zakikhany, K., C. R. Harrington, M. Nimtz, J. C. Hinton, and U. Romling.** 2010. Unphosphorylated CsgD controls biofilm formation in *Salmonella enterica serovar Typhimurium*. Mol Microbiol.

74. **Zhang, X. S., R. Garcia-Contreras, and T. K. Wood.** 2007. YcfR (BhsA) influences *Escherichia coli* biofilm formation through stress response and surface hydrophobicity. J Bacteriol **189:**3051-62.

75. **Zogaj, X., M. Nimtz, M. Rohde, W. Bokranz, and U. Romling.** 2001. The multicellular morphotypes of *Salmonella typhimurium* and *Escherichia coli* produce cellulose as the second component of the extracellular matrix. Mol Microbiol **39:**1452-63.
